# From common gardens to candidate genes: exploring local adaptation to climate in red spruce

**DOI:** 10.1111/nph.18465

**Published:** 2022-10-13

**Authors:** Thibaut Capblancq, Susanne Lachmuth, Matthew C. Fitzpatrick, Stephen R. Keller

**Affiliations:** ^1^ Department of Plant Biology University of Vermont Burlington VT 05405 USA; ^2^ Appalachian Laboratory University of Maryland Center for Environmental Science Frostburg MD 21532 USA

**Keywords:** adaptive genes, climate transfer distance, conifer, genome scan, genotype‐environment association, *Picea rubens*

## Abstract

- Local adaptation to climate is common in plant species and has been studied in a range of contexts, from improving crop yields to predicting population maladaptation to future conditions. The genomic era has brought new tools to study this process, which was historically explored through common garden experiments.
- In this study, we combine genomic methods and common gardens to investigate local adaptation in red spruce and identify environmental gradients and loci involved in climate adaptation. We first use climate transfer functions to estimate the impact of climate change on seedling performance in three common gardens. We then explore the use of multivariate gene–environment association methods to identify genes underlying climate adaptation, with particular attention to the implications of conducting genome scans with and without correction for neutral population structure.
- This integrative approach uncovered phenotypic evidence of local adaptation to climate and identified a set of putatively adaptive genes, some of which are involved in three main adaptive pathways found in other temperate and boreal coniferous species: drought tolerance, cold hardiness, and phenology. These putatively adaptive genes segregated into two ‘modules’ associated with different environmental gradients.
- This study nicely exemplifies the multivariate dimension of adaptation to climate in trees.

## Abstract 2

See also the Commentary on this article by Eckert & Neale, **237**: 1479–1482.

## Introduction

Local adaptation arises when different populations of the same species genetically diverge to produce phenotypes that maximize their fitness in their local environment, regardless of the efficiency of these phenotypes in other environments (Kawecki & Ebert, 2004). This process is commonly encountered in plants (Leimu & Fischer, 2008) and has been extensively studied in trees (Savolainen *et al*., 2007; Sork, 2018), which are often distributed across large climatic gradients that enhance divergent selection that shapes local adaptation. Trees also frequently are foundational species, structuring the habitat and its associated ecosystem (Ellison *et al*., 2005), and are of important agricultural and economical value. Motivations for studying local adaptation in trees thus include enhancing plantation productivity (Wang *et al*., 2010), selecting optimal source populations (Steane *et al*., 2014), and restoring disturbed habitats (Prober *et al*., 2015). More recently, studies have considered local adaptation when evaluating tree species capacity to respond to ongoing climate change (Alberto *et al*., 2013), including assessments of population adaptability (Visser, 2008; Hoffmann & Sgró, 2011), future maladaptation (Fitzpatrick & Keller, 2015) and the feasibility of assisted migration (Aitken *et al*., 2008; Aitken & Bemmels, 2016; Browne *et al*., 2019).

Given that locally adapted genotypes should have higher fitness in their native environment than genotypes originating from other environments (Kawecki & Ebert, 2004), a good way to detect local adaptation is to transfer individuals from various environmental sources (a.k.a. provenances) into a new environment (i.e. common garden) and assess whether local individuals show higher fitness than foreign ones (Blanquart *et al*., 2013; Lascoux *et al*., 2016). Common gardens (a.k.a. provenance trials) have become powerful tools in both applied and basic research to study tree adaptation to climate (Langlet, 1971; Savolainen & Pyhäjärvi, 2007; Browne *et al*., 2019). Recently, local adaptation has also increasingly been investigated using large genomic datasets (Savolainen *et al*., 2013; Whitlock, 2015), either to identify genes involved in the divergent expression of known adaptive traits (Wadgymar *et al*., 2017) or to examine the association between genetic variation and environmental gradients in a landscape genomics framework (Sork *et al*., 2013; Tiffin & Ross‐Ibarra, 2014; Hoban *et al*., 2016; Martins *et al*., 2018). Exploring the geographic distribution of adaptive alleles also yields valuable information on species adaptation strategies and associated landscape constraints (Steane *et al*., 2014). Recent studies show that different groups of loci involved in local adaptation to climate can vary in different ways along environmental gradients or at different spatial scales in forest tree species (Mahony *et al*., 2020; Gugger *et al*., 2021).

Finding the genes underlying local adaptation is largely achieved using genome scan approaches belonging to the genotype–environment association (GEA) family (Hoban *et al*., 2016). Here, an important recent development is the use of multivariate approaches that avoid problems with multiple testing of the same loci in response to different predictors and allow identification of complex environmental gradients that drive adaptation (Fitzpatrick & Keller, 2015; Forester *et al*., 2018). Another challenge of GEA methods is to distinguish the genomic patterns left by demographic history from those resulting from environmental selection, which often covary and blur the search for selection (Frichot *et al*., 2015; Whitlock & Lotterhos, 2015; Hoban *et al*., 2016). A common approach to avoid false positives due to demographic history is to account for population structure by conditioning the models with measures of neutral genetic variation as covariates (Tibbs Cortes *et al*., 2021). Nonetheless, if neutral genetic variation strongly covaries with environmental gradients, then removing the statistical signal associated with neutral variation is likely to also remove signals associated with environmental selection (Savolainen *et al*., 2013), leading to a higher frequency of false negatives and failure to detect locally adapted variants (Sork *et al*., 2013; Ahrens *et al*., 2021). The field of ecological genomics still struggles with how to identify genes underlying local adaptation when demographic and environmental gradients strongly covary, as is the case for many temperate species that experienced range contractions and expansions oriented along latitudinal gradients during previous glacial cycles (Hewitt, 1999).

One such species is red spruce (*Picea rubens* Sarg.) – a temperate conifer endemic to north‐eastern America with a distribution that spans 13° of latitude, and 800 m of elevation (Verrico *et al*., 2019), making the species a good candidate for climatically‐driven divergent selection. However, as red spruce colonized its current range from a southern refugium after the Last Glacial Maximum (LGM), it followed a south–north expansion route (T. Capblancq *et al*., unpublished) such that the climatic gradients that drive local adaptation strongly covary with neutral population structure and geographic distance. As such, the species provides an interesting case for disentangling those confounded factors and for exploring the outcomes of multivariate GEA approaches.

Here, we tested for local adaptation to climate in red spruce and explored associated genetic and abiotic factors across the species range. We first analyzed the influence of climate transfer distance on seedling performance in three common gardens and found phenotypic evidence of adaptation to climate, confirming the presence of local adaptation in red spruce and supporting the search for associated adaptive genes. Then, to identify genomic signatures of local adaptation, we used multivariate GEA methods and compared the results obtained when correcting or not for neutral population structure. By investigating the function and variation of the loci that were most strongly associated with climate gradients, we identified putatively adaptive genes involved in: response to drought or cold; regulation of flowering time; and mechanisms of DNA and RNA repair. These candidate genes clustered into two different ‘modules’ based on their variation in allele frequencies along climatic gradients, resulting in markedly different trajectories of spatial turnover across geographic and climatic landscapes. Despite some challenges posed by the demographic history of red spruce, our results revealed the occurrence and underlying genetic basis of local adaptation to climate in this important tree species. Our study improves understanding of the process of local adaptation in conifer tree species and opens the door to future genetically informed measures for managing red spruce adaptation under environmental change.

## Materials and Methods

### Establishing the presence of local adaptation

**Fig. 1:**
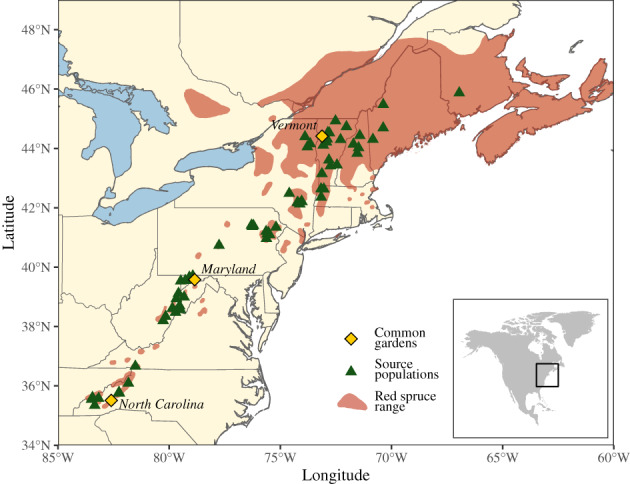
Geographic distribution of red spruce *Picea rubens* in eastern North America (according to Little Jr., 1971) with the locations of the 65 source localities and the three common garden sites analyzed in this study.

To assess the presence of local adaptation in red spruce, we tested the influence of climate transfer distance on seedling early‐life performance at three different common garden sites. 1700 seedlings from 340 families (single mother trees) and 65 localities were grown in raised beds at three locations: Ashville, North Carolina, Frostburg, Maryland, and Burlington, Vermont, USA (Fig. 1; Supporting Information Table S1).

The experiments followed a randomized block design with five blocks/site and one seedling/family/block, leading to 5100 seedlings experiment‐wide (1700 seedlings/site × 3 sites). Seedlings were germinated in spring 2018 and raised in a glasshouse at the University of Vermont before being planted in the gardens in June 2019 (*c*. 1 yr old). The experiment ran for two complete growing seasons and one winter season, ending in October 2020. Seedling height was recorded at the beginning and end of the experiment, and we used the increment – Height Growth – as a proxy of seedling early‐life performance (dead seedlings were scored zero). An estimate of trait value for each family was produced using the BLUPs (best linear unbiased predictors) of a linear mixed model that included a combined bed‐rack variable (five beds/garden and five racks/bed = 25 bed‐racks/garden) and family (340 seedling mother trees) as random effects, independently for each garden.

To assess phenotypic evidence of local adaptation, we tested for the predicted negative relationship between Height Growth and climate transfer distance estimated between each source locality and the gardens. We selected 11 variables that captured the climatic niche of our sampling of red spruce's distribution, while exhibiting minimal collinearity and characterizing biologically relevant environments for adaptation in trees (Fig. S1). These variables were: degree days below 0°C (DD_0), degree days above 18°C (DD18), mean annual solar radiation (MAR), precipitation as snow (PAS), May to September precipitation (MSP), mean annual relative humidity (RH), extreme maximum temperature (EXT), climatic moisture deficit (CMD), Continentality (TD), end of the frost free period (eFFP), potential evapo‐transpiration (PET). All variables came from the climateNA database (Wang *et al*., 2016) except PET, which was obtained from the ENVIREM database (Title & Bemmels, 2018).

We used climate normals for 1961–1990 to describe the climate of source localities and values for 2019 and 2020 to describe climate at the common garden sites. A climate transfer distance – the difference between the climate normals at a source locality and the climate experienced at a common garden site – was estimated between each source locality/garden pair using a multivariate approach. A principal component analysis (PCA) was conducted on a standardized matrix including the climatic values of source localities and garden sites together. We estimated Euclidean distances between each source locality and the three gardens using all 11 principal components (PCs). We then tested the association between climate transfer distance and mean Height Growth estimated for each source locality using linear and quadratic regressions. We hypothesized that, if red spruce exhibited local adaptation to climate, an increased climate transfer distance would negatively affect seedling fitness in the common gardens.

### Genomic data acquisition

We used population genomic data from a previously published whole‐exome capture sequencing experiment conducted on the 340 mother trees described earlier (Table S1) (Capblancq *et al*., 2020a). Since no reference genome for *P. rubens* is available, we mapped the sequence reads against the Norway spruce (*Picea abies*) reference genome (Nystedt *et al*., 2013). We used ANGSD (Analysis of Next Generation Sequencing Data) (Korneliussen *et al*., 2014), to produce genotype likelihoods for each individual and covered genomic site, accounting for uncertainty associated with the low coverage of these data (mean coverage = 2.28 reads/individual/site). We followed the same bioinformatic protocol and filtering options as in Capblancq *et al*. (2020a) except that we mapped to Norway spruce instead of the white spruce (*Picea glauca*) reference genome to take advantage of the former's information on functional annotation (available at www.congenie.org). One individual having poor quality sequences and seven individuals identified as artificially transplanted trees from different locations (Capblancq *et al*., 2020a) were removed from the dataset, leaving a final genomic dataset of 332 individuals from 64 localities and 917 234 single nucleotide polymorphisms (SNPs).

We used *pcangsd* (Meisner & Albrechtsen, 2018) to produce, from genotype likelihoods, a posterior expectation of the genotype ranging continuously from 0 to 2, called genotype dosage, for each polymorphic site and individual. During this step we also filtered loci that had a minor allele frequency below 10% across the complete sampling, leaving 335 588 loci in the filtered dataset. The genotype dosages were averaged and divided by two within each of the 64 localities to estimate minor allele frequencies. We also used *pcangsd* to produce a genetic covariance matrix for the 332 individuals, which we used to find the loadings of the individuals along the different PCs characterizing the genetic variation across the sampling. The loadings of localities along the first two genetic PCs were used as conditioning variables in downstream analyses to account for population structure.

### Exploring the drivers of genetic variation

To estimate the relative influence of environment, geography and demographic history in driving genetic variation across red spruce populations, we conducted a series of partial redundancy analyses (pRDAs). We conducted the pRDAs using three groups of variables. First, we used locality geographic coordinates to produce distance‐based Moran's Eigenvector Maps (dbMEMs) and kept the first three dbMEMs as proxies of geographic structure across sampling sites (Legendre & Legendre, 2012). Second, we used loadings along genetic PC1 and PC2 (see earlier), as proxies of neutral genetic structure. Third, we used the 11 selected climate variables to characterize environmental variation across the sampled localities. Variance partitioning was carried out using locality allele frequencies as the response variable in different models: a full model using all variables as explanatory variables, and three partial models using either the 11 climate variables, the three dbMEMs or the two genetic PCs as explanatory variables and the other variables as conditioning variables. The full model returned the total amount of variance (a.k.a. inertia) explained by climate, geography and neutral genetic structure together while each of the pure models returned the unique portion of variance explained by each one of these factors after conditioning on the remaining variables. The analyses were conducted using the function *rda* of the R‐package vegan (Oksanen *et al*., 2015).

### Detection of adaptive loci

We searched for loci covarying strongly with climatic gradients using two multivariate GEA methods: a genome scan approach based on redundancy analysis (RDA) described in Capblancq *et al*. (2018), and the genome scan approach described in Fitzpatrick *et al*. (2021) based on Gradient Forest (GF) models using either raw allele frequencies (GF‐raw) or allele frequencies after correction for population relatedness (GF‐X). In brief, the RDA‐based method rearranges redundant genetic variation associated with environmental variation along composite axes and identifies the loci that are strongly associated with the most important axes (Capblancq *et al*., 2018). The GF‐based approaches use the machine‐learning random forest algorithm to evaluate how much of the among‐population variance in allele frequencies at a locus is explained by a set of environmental variables, summarized as *R* ^2^ (Fitzpatrick *et al*., 2021). Both RDA and GF carry an assumption of monotonicity; however, RDA assumes that the relationship between genetic and environmental variation is linear whereas GF is nonparametric and makes no assumption about the particular form of the gene–environment relationship. Both methods were conducted using population (e.g. locality) allele frequencies obtained from the genotype dosages and our 11 climate variables. To test if these multivariate methods departed from established univariate methods, we repeated these analyses using Bayenv2 (Günther & Coop, 2013) and lfmm (Frichot *et al*., 2013).

As described in the Introduction, it is common to account for population structure during a genome scan analysis to minimize false positives arising due to neutral loci that show allele frequency variance among populations. However, correcting for population structure could artificially increase false negatives and result in a failure to detect true positive loci under selection. This trade‐off becomes especially problematic for species like red spruce in which the expected drivers of climate‐driven selection are strongly collinear with geography and neutral genetic structure. To explore this issue, the RDA was performed either with no correction for population structure (hereafter RDA‐raw) or using the averaged locality scores on the first two axes of the genetic PCA as conditioning variables (hereafter RDA‐corrected). Similarly, GF was conducted on raw locality allele frequencies (hereafter GF‐raw) and on standardized allele frequencies corrected for population relatedness (hereafter GF‐X; Fitzpatrick *et al*., 2021) as obtained from bayenv2 (Günther & Coop, 2013) and a population allele frequency (co)variance matrix (‘omega’) estimated using 100 000 iterations of the MCMC chain. We then used the omega matrix to obtain estimates of the standardized allele frequencies using the ‘‐f’ flag, and saved 190 draws from the posterior distribution to integrate over uncertainty in the estimates.

Finally, to identify loci that were putatively under selection by climate, we rank‐ordered loci and identified the top 0.2% (i.e. 671 loci) from each method's statistics – Mahalanobis distances from RDA (RDA‐raw and RDA‐corrected) and *R* ^2^ from GF (GF‐raw and GF‐X) – and retained loci common to at least two of the four genome scans. Following (Coop *et al*., 2010; Yoder *et al*., 2014), we chose a rank‐based assessment of outliers, because the GF‐raw and GF‐X method does not produce *P*‐values, and because significance thresholds in genome scans are always somewhat arbitrary. Our goal here was to obtain a set of loci that was enriched for loci involved in adaptation to climate. We also tested the link between seedling early‐life performance and genotypic variation using linear mixed models with Height Growth as response variable, individual genotypes (coded as semi‐quantitative variable: 0/1/2) as fixed effect and source locality as a random effect, independently for each common garden site and for each of the 335 588 loci. We hypothesized that Height Growth, as a proxy of fitness, should be better explained by a set putatively adaptive loci as compared to nonadaptive loci, which we tested comparing *R* ^2^ values of the two groups of loci using a Wilcoxon–Mann–Whitney test. We used the R‐packages ‘lme4’ (Bates *et al*., 2015) and ‘r2glmm’ (Johnson, 2014) to complete the different analyses.

### Functional annotation and ontology of candidate loci

We used SNPeff (Cingolani *et al*., 2012) and the positions of the putatively adaptive loci (i.e. selected during the genome scan analyses) along the *P. abies* reference genome to annotate adaptive variants to functional classes: upstream and downstream of genes, introns, synonymous, nonsynonymous, intronic, or intergenic sites. When the markers fell within or nearby (< 5 k base pairs up or downstream) known genes we used the ConGenIE suite (https://congenie.org, Sundell *et al*., 2015) to find the gene's functional annotation and look for their involvement in adaptation in other species. Finally, to assess if the list of annotated candidate genes were overrepresented for particular molecular functions or biological processes, we conducted a gene ontology (GO) term enrichment analysis using the ConGenIE online GO enrichment tool.

### Distribution of adaptive variation across the landscape

To map the climatic and spatial distribution of adaptive genetic variation, we conducted an RDA using raw locality allele frequencies from only putatively adaptive loci as response variables and the 11 climatic variables as explanatory variables. We then retrieved from this adaptively‐enriched RDA the loadings of each environmental predictor and used those scores to predict an adaptive genetic index for each pixel of the range, as described in Capblancq *et al*. (2020b). Different ‘modules’ of outlier loci were also identified based on their positions along the adaptively enriched RDA axes using a Euclidean *k*‐means clustering algorithm, implemented in the R‐package stats (R Core Team, 2020). We used the absolute value of the adaptive loci scores on RDA1 and RDA2 to identify two clusters representing (1) loci particularly associated with RDA1 (i.e. high absolute RDA1 score and low RDA2 score) and (2) loci particularly associated with RDA2 (i.e. high absolute RDA2 score and low RDA1 score).

## Results

### Seedling response to climate transfer in the common gardens

**Fig. 2:**
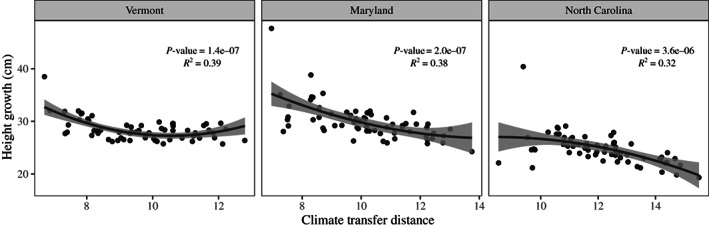
Association between climate transfer distance based on 11 variables and seedling fitness represented by mean height growth per source locality at the three red spruce common garden sites. The significance of the association was tested in each garden using a quadratic regression, the resulting *P*‐values for the quadratic term and *R* ^2^ are shown on each panel. Lines show model predictions and the gray areas show the 95% confidence intervals.

Red spruce showed significant evidence of local adaptation based on phenotypic performance in the common gardens. We found a significant negative association between seedling growth and climate transfer distance in all three gardens (Fig. 2), with quadratic regressions returning *R* ^2^ values of 0.39 in Vermont, 0.38 in Maryland and 0.32 in North Carolina. Linear regressions were also highly significant, with *R* ^2^ ranging from 0.21 to 0.37 (Fig. S2). The decrease in Height Growth between source localities experiencing the smallest climate transfer and those experiencing the largest climate transfer ranged from *c*. 3 cm in Vermont to *c*. 10 cm in Maryland (Fig. 2). Note here that climate transfer distance (i.e. the difference in climate between a source locality and a common garden site) is estimated using a multivariate approach based on a climatic PCA and thus only returns positive values, as opposed to univariate transfer distance which can be negative if the garden site climate value is higher than the one at the source locality.

### Strong confounding effect of genetic structure and geographic distance on the genetic–environment relationship

We observed a high degree of explanatory power in pRDA models predicting allele frequencies from geography, neutral genetic structure, and climate, but also a significant amount of confounding between all three groups of predictors. Together, climate, geography and neutral structure explained 41% of variance in allele frequencies among red spruce localities (Table 1). After conditioning allele frequencies on neutral genetic structure and geography, the pure effect of climate was no longer significant, but did explain 14% of the total genetic variation (34% of the variation that was explainable by the full model). The pure effect of neutral genetic structure was significant and explained 3% of total genetic variance (8% of explainable variation) while geography significantly explained 5% (12% of explainable variation). Interestingly, we found that 45% of explained genetic variance was confounded among the different groups of predictors, and reflected the high degree of collinearity between climate, geography and neutral genetic structure (Fig. S3).

**Table 1:** The influence of climate, geography and neutral genetic structure on red spruce genetic variation was decomposed through a series of partial redundancy analyses (pRDAs).

Partial RDA models	Inertia	*P* (> *F*)	Proportion of explainable inertia	Proportion of total inertia
Full model: *F ~ clim. + struc. + geog*.	1154.8	0.001***	1	0.41 (*R* ^2^)
Pure climate: *F ~ clim. |* (*geog. + struc*.)	394.5	0.289	0.34	0.14 (*R* ^2^)
Pure geography: *F ~ geog. |* (*clim. + struc*.)	143.1	0.001***	0.12	0.05 (*R* ^2^)
Pure ancestry: *F ~ struc. |* (*clim. + geog*.)	94.3	0.001***	0.08	0.03 (*R* ^2^)
Confounded climate/geography/structure	523.0		0.45	0.19
Total unexplained	1657.8			0.59
Total inertia	2812.6			1.00

The statistical significance is given for each model (***, *P* ≤ 0.001) together with the percentage of explained genetic variance compared to the variance explained by the full model and compared to the total variance present in the dataset. The proportion of total variance explained given in the last column corresponds to the *R* ^2^ of the regression for the tested models.

### Genomic signature of adaptation to climate

**Fig. 3:**
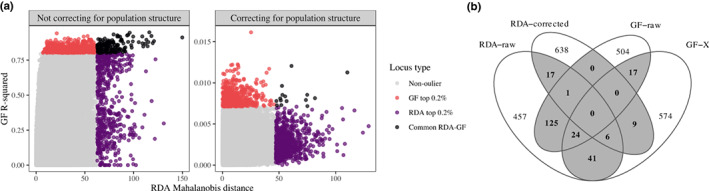
Results of the genome scans with (a) the correspondence between redundancy analysis (RDA) and gradient forest (GF) methods when using raw allele frequencies (left panel) or allele frequencies corrected for population structure (right panel), and (b) a Venn diagram showing the overlap of the top 0.2% loci identified with each procedure. Note the large difference in *y*‐axis scale between the two panels of (a).

A total of 335 588 loci exhibited a minor allele frequency > 0.1 and were included in the multivariate GEAs. We observed substantial overlap between RDA and GF when neutral population structure was not accounted for, with 149 loci (i.e. 22% of each set) found by both methods (Figs 3, S4). On the contrary, congruence was low when correcting for population structure, with only 15 common loci (2%) between methods, and no visible covariation between GF *R* ^2^ and RDA Mahalanobis distance (Fig. 3a). Noticeably, accounting for population structure substantially reduced the variance in allele frequencies explained by climate using GF, which reached a maximum of *R* ^2^ = 0.954 when modeling raw allele frequencies (i.e. GF‐raw) but only a maximum of *R* ^2^ = 0.016 when modeling allele frequencies corrected for population structure with bayenv2 (i.e. GF‐X). The loci identified when accounting or not for population structure showed little overlap between the two RDA genome scans (24/1342 loci) and between the two GF genome scans (41/1342 loci) (Fig. 3b). For downstream analyses, we considered as putative adaptive loci the 240 loci in the top 0.2% for at least two of the four genome scan procedures (Fig. 3b). When we compared the proportion of genetic variance explained by the 11 climate variables for the four sets of loci identified as outliers by each method, we observed that they showed the expected association with climate variation (0.43 < *R* ^2^ < 0.84), more so than a corresponding random set of loci (*R* ^2^ = 0.31) (Table S2). The 240 putatively adaptive loci had the second highest *R* ^2^ (0.81), just below the set of loci identified with GF‐raw (*R* ^2^ = 0.84) and above the RDA‐raw set of loci (*R* ^2^ = 0.71). The *R* ^2^ values obtained with loci identified by GF‐X and RDA‐corrected (0.62 and 0.43, respectively) were lower than those obtained for GF‐raw and RDA‐raw but higher than the random set (*R* ^2^ = 0.31).

The results of the multivariate genome scans partially overlapped with the top loci identified using lfmm and bayenv2 univariate methods (Fig. S5). The greatest overlap was between lfmm and RDA‐raw (204 loci) and GF‐raw (268 loci). Interestingly, there was greater overlap between the multivariate and univariate methods than between Bayenv2 and lfmm (179 loci), even though many more loci were considered for these two univariate scans due to the multiplication of the top 0.2% loci set (i.e. one per environmental variable, *N* = 6606 for Bayenv2 and 5604 for lfmm). To a smaller extent, lfmm results also overlapped with RDA‐corrected (104 loci) or GF‐X (65 loci) while Bayenv2 results were less congruent with RDA or GF, corrected or not, with only 16 to 51 loci in common (Fig. S5).

**Fig. 4:**
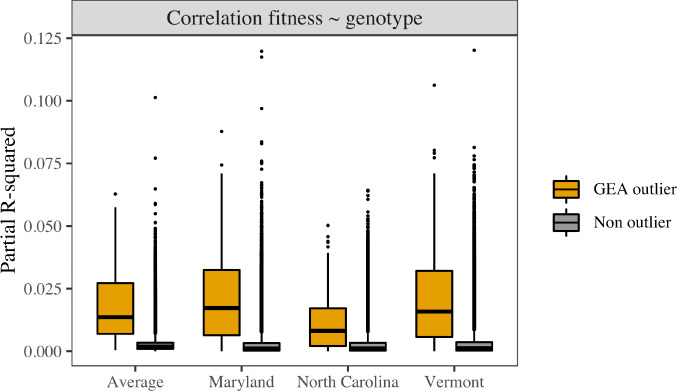
Explanatory power of linear mixed models that regressed height growth in the red spruce common gardens against genotypes and used source locality as random effect. The distribution of genotype fixed effect partial *R* ^2^ values is shown for each garden and averaged across all three garden sites. The 240 loci identified as putatively under selection for climate (i.e. genome scan outliers) were compared to the nonoutlier loci using Wilcoxon–Mann–Whitney test: highly significant differences (*P*‐value << 0.001) were found at each garden site as well as for the average. The boxplot black line shows the median of the distribution when the colored box frames the values between the lower and upper quartiles and the outlier points correspond to values beyond the lower quartile − 1.5 × interquartile range and the upper quartile + 1.5 × interquartile range.

The relationship between seedling early‐life fitness, represented by Height Growth, and candidate loci for climate adaptation was consistent with polygenic adaptation across each of the three common gardens (Fig. 4). Individual GEA outlier loci explained *c*. 1–3% of phenotypic variance on average, which was significantly higher compared to nonoutlier loci (< 0.01% on average).

### Molecular pathways and genes associated with local adaptation in red spruce

We found that most of the 240 putatively adaptive loci (147/240–61.3%) fell within or close (< 5 kb) to 125 known genes (Table S3). Among these 125 candidate genes, 105 were annotated as protein‐coding with a proposed function (Table 2). Many of these genes are known to be involved in other species' adaptation to climatic gradients. In particular, our list of annotated candidate genes included *At4g33300*, involved in drought resistance and defense response in *Abies alba* (Behringer *et al*., 2015) and *Arabidopsis thaliana* (Bonardi *et al*., 2017); *NBR1* which is involved in response to heat and drought stress in *Arabidopsis thaliana* (Zhou *et al*., 2013); *HSFB‐2B*, commonly involved in heat stress regulation in plants (Guo *et al*., 2016); *FPA*, that regulates flowering time in *Arabidopsis thaliana* via a pathway that is independent of daylength (Schomburg *et al*., 2001); a ribosomal RNA (rRNA) methylase known to play a role in response to stress by enhancing or reducing translation of specific messenger RNAs (mRNAs) (Liberman *et al*., 2020); *DNA damage‐binding 1*, involved in *Brassicaceae* adaptation to high altitude (Guo *et al*., 2018); *DHAR2*, involved in ascorbate production, which mitigates reactive oxygen species (ROS) free radicals produced in response to abiotic stress in *Arabidopsis thaliana* (Terai *et al*., 2020). A complete list of genes and their involvement in adaptation to climate in other plant species is given in the Notes S1.

**Table 2:** List of the 125 genes associated with the putatively adaptive loci identified by the genome scans in red spruce, including gene names, a description of the associated protein functions, the number of loci identified as outliers for each gene, the gene cluster they belong to based on redundancy analysis (RDA) scores and the genotype–environment association (GEA) methods that detected the underlying loci.

Gene	Description	Chromosome	Confidence	Cluster	Nb loci	GEA method
MA_10227813g0010	Cysteine and histidine‐rich domain‐containing RAR1	MA_10227813	High	1	1	RDA‐corr/GF‐X
MA_103156g0020	VIN3 1	MA_103156	High	1	2	RDA‐raw/GF‐raw
MA_10425950g0010	Probable carotenoid cleavage dioxygenase chloroplastic	MA_10425950	High	1	1	GF‐raw/GF‐X
MA_10426447g0010	Rac‐like GTP‐binding 5	MA_10426447	High	1	2	RDA‐raw/GF‐raw/GF‐X
MA_10427427g0010	Beta‐galactosidase 5‐like	MA_10427427	High	1	1	RDA‐raw/GF‐raw
MA_10429414g0020	Ubiquitin carboxyl‐terminal hydrolase 18‐like	MA_10429414	High	1	1	RDA‐raw/GF‐raw
MA_10429519g0010	Probable disease resistance At4g33300	MA_10429519	High	1	2	RDA‐raw/GF‐raw
MA_10429540g0010	Pentatricopeptide repeat‐containing At4g14850	MA_10429540	High	1	1	RDA‐raw/GF‐raw/GF‐X
MA_10430323g0010	Polyadenylate‐binding RBP47B	MA_10430323	High	1	2	RDA‐raw/GF‐raw
MA_10431352g0010	PREDICTED: uncharacterized protein LOC104601792 isoform X2	MA_10431352	High	1	1	RDA‐raw/GF‐X
MA_10432774g0010	E3 ubiquitin‐ ligase listerin	MA_10432774	High	1	1	RDA‐raw/GF‐raw
MA_10434569g0010	Glycerol‐3‐phosphate dehydrogenase (NAD+) cytosolic‐like	MA_10434569	High	1	1	RDA‐raw/GF‐raw
MA_10434999g0010	DDB1‐ and CUL4‐associated factor 8	MA_10434999	High	1	1	RDA‐raw/GF‐raw
MA_10435282g0010	Zinc finger CCCH domain‐containing ZFN‐like isoform X2	MA_10435282	High	1	1	RDA‐raw/GF‐raw/GF‐X
MA_10435314g0010	Flowering time control FPA	MA_10435314	High	1	1	RDA‐raw/GF‐raw
MA_10436174g0010	NBR1 homolog	MA_10436174	High	1	1	RDA‐raw/GF‐raw
MA_10437228g0010	Probable cyclic nucleotide‐gated ion channel chloroplastic	MA_10437228	High	1	1	RDA‐raw/GF‐raw
MA_107207g0010	RETICULATA‐RELATED chloroplastic‐like	MA_107207	High	1	1	RDA‐raw/GF‐raw
MA_11235g0010	Unknown	MA_11235	High	1	1	RDA‐raw/GF‐X
MA_118674g0010	Probable galacturonosyltransferase 13 isoform X1	MA_118674	High	1	2	RDA‐raw/RDA‐corr/GF‐raw
MA_122077g0010	Chloroplastic	MA_122077	High	1	2	RDA‐raw/GF‐raw
MA_12771g0010	Importin subunit beta‐1	MA_12771	High	1	1	RDA‐raw/GF‐raw
MA_128244g0010	Myb‐related Zm38‐like	MA_128244	High	1	1	RDA‐raw/GF‐raw
MA_130934g0010	Probable galacturonosyltransferase‐like 3	MA_130934	High	1	1	RDA‐raw/GF‐raw
MA_132866g0010	Probable ubiquitin‐conjugating enzyme E2 18	MA_132866	High	1	1	RDA‐corr/GF‐X
MA_13800g0010	na	MA_13800	High	1	1	RDA‐raw/GF‐raw
MA_14836g0010	TRANSPORT INHIBITOR RESPONSE 1‐like	MA_14836	High	1	1	RDA‐raw/GF‐raw
MA_177060g0010	na	MA_177060	High	1	1	RDA‐raw/GF‐raw
MA_180963g0010	Rare cold inducible	MA_180963	High	1	1	RDA‐raw/GF‐raw
MA_18142g0010	Serine–glyoxylate aminotransferase	MA_18142	High	1	1	RDA‐raw/GF‐raw/GF‐X
MA_1829g0010	Aspartate carbamoyltransferase chloroplastic	MA_1829	High	1	1	RDA‐raw/GF‐raw
MA_20599g0020	Pentatricopeptide repeat‐containing At2g13600‐like	MA_20599	High	1	1	RDA‐raw/RDA‐corr
MA_21020g0010	Plastid division PDV1	MA_21020	High	1	1	RDA‐raw/GF‐raw
MA_30433g0010	DUF179 domain‐containing	MA_30433	High	1	2	RDA‐raw/GF‐raw/GF‐X
MA_3259g0010	Coatomer subunit epsilon‐1	MA_3259	High	1	1	RDA‐raw/GF‐raw
MA_3352g0010	Transcription factor PCL1	MA_3352	High	1	1	RDA‐raw/GF‐X
MA_3745g0010	ALA‐interacting subunit 3	MA_3745	High	1	4	RDA‐raw/GF‐raw/GF‐X
MA_37500g0010	Unknown	MA_37500	High	1	1	RDA‐raw/GF‐raw
MA_3809g0010	Zinc finger CCHC domain‐containing 8 isoform X2	MA_3809	High	1	1	RDA‐raw/GF‐raw
MA_415377g0010	Transcription termination factor chloroplastic‐like	MA_415377	High	1	1	RDA‐raw/RDA‐corr
MA_45528g0010	Probable calcium‐binding CML49	MA_45528	High	1	2	RDA‐raw/RDA‐corr/GF‐X
MA_46875g0010	Pentatricopeptide repeat‐containing At1g20230	MA_46875	High	1	1	RDA‐corr/GF‐X
MA_5374g0010	Heat stress transcription factor B‐2b‐like	MA_5374	High	1	2	RDA‐raw/GF‐raw
MA_571234g0010	Unknown	MA_571234	High	1	1	RDA‐raw/GF‐raw
MA_60015g0010	PREDICTED: uncharacterized protein LOC103336100	MA_60015	High	1	1	RDA‐raw/GF‐raw
MA_60789g0010	PREDICTED: uncharacterized protein LOC103724222	MA_60789	High	1	1	RDA‐raw/GF‐raw/GF‐X
MA_6619g0010	Cyclin‐A1‐4 isoform X1	MA_6619	High	1	1	GF‐raw/GF‐X
MA_6634g0010	DNA topoisomerase 2	MA_6634	High	1	1	RDA‐corr/GF‐X
MA_735g0010	BP28CT domain‐containing U3snoRNP10 domain‐containing	MA_735	High	1	1	RDA‐raw/GF‐raw/GF‐X
MA_74926g0010	Carboxyl‐terminal‐processing peptidase chloroplastic	MA_74926	High	1	1	RDA‐raw/GF‐raw
MA_78965g0010	Peptidyl‐prolyl cis‐trans isomerase Pin1	MA_78965	High	1	1	GF‐raw/GF‐X
MA_83834g0010	Hypothetical protein L484_013434	MA_83834	High	1	2	RDA‐raw/GF‐raw/GF‐X
MA_88112g0010	E3 ubiquitin‐ ligase MARCH8‐like isoform X1	MA_88112	High	1	1	RDA‐raw/GF‐raw/GF‐X
MA_88535g0010	Probable galacturonosyltransferase‐like 7	MA_88535	High	1	1	RDA‐raw/GF‐raw
MA_8859g0020	na	MA_8859	High	1	1	RDA‐raw/GF‐raw
MA_88915g0010	Probable E3 ubiquitin‐ ligase ARI8	MA_88915	High	1	1	RDA‐raw/GF‐raw
MA_9019430g0010	na	MA_9019430	High	1	1	RDA‐raw/GF‐raw
MA_94664g0010	PREDICTED: uncharacterized protein LOC107412951 isoform X1	MA_94664	High	1	1	GF‐raw/GF‐X
MA_97731g0010	Katanin p80 WD40 repeat‐containing subunit B1 homolog isoform X1	MA_97731	High	1	1	RDA‐corr/GF‐X
MA_98067g0010	Pentatricopeptide repeat‐containing At5g04780‐like	MA_98067	High	1	1	RDA‐raw/GF‐raw
MA_9905g0010	2–3 ethylene‐responsive transcription factor	MA_9905	High	1	1	RDA‐raw/GF‐raw
MA_10270424g0010	Probable tRNA N6‐adenosine mitochondrial isoform X2	MA_10270424	Low	1	1	RDA‐raw/GF‐raw
MA_10427318g0010	na	MA_10427318	Low	1	1	RDA‐raw/GF‐raw/GF‐X
MA_110335g0010	na	MA_110335	Low	1	1	RDA‐raw/GF‐raw/GF‐X
MA_181884g0020	na	MA_181884	Low	1	2	RDA‐raw/GF‐raw/GF‐X
MA_491983g0010	Unknown	MA_491983	Low	1	1	GF‐raw/GF‐X
MA_50524g0010	Lactosylceramide 4‐alpha‐galactosyltransferase‐like	MA_50524	Low	1	3	RDA‐raw/GF‐raw
MA_10045090g0010	NAD(P)‐binding rossmann‐fold	MA_10045090	Medium	1	1	RDA‐raw/GF‐raw
MA_10182517g0010	Methyltransferase 6	MA_10182517	Medium	1	2	RDA‐raw/GF‐raw/GF‐X
MA_10261150g0010	PREDICTED: uncharacterized protein LOC102625808 isoform X2	MA_10261150	Medium	1	1	RDA‐raw/GF‐raw
MA_10427896g0020	Signal recognition particle 19 kDa	MA_10427896	Medium	1	2	RDA‐raw/GF‐X
MA_10430375g0020	Cytochrome b5 domain‐containing RLF	MA_10430375	Medium	1	1	RDA‐raw/GF‐raw
MA_10431074g0010	Transmembrane 161B	MA_10431074	Medium	1	1	RDA‐raw/GF‐raw
MA_10432806g0020	LAG1 longevity assurance homolog 3‐like	MA_10432806	Medium	1	1	RDA‐raw/GF‐raw
MA_10433025g0010	tRNA wybutosine‐synthesizing 2 3 4 isoform X1	MA_10433025	Medium	1	1	GF‐raw/GF‐X
MA_10433880g0010	SUPPRESSOR OF npr1‐ CONSTITUTIVE 1‐like isoform X2	MA_10433880	Medium	1	1	RDA‐raw/GF‐raw/GF‐X
MA_10434616g0010	Pentatricopeptide repeat‐containing mitochondrial	MA_10434616	Medium	1	1	RDA‐raw/RDA‐corr
MA_10435503g0020	U‐box domain‐containing 44‐like	MA_10435503	Medium	1	1	RDA‐raw/GF‐raw
MA_10436633g0020	Disease resistance RPP13 4	MA_10436633	Medium	1	1	GF‐raw/GF‐X
MA_10437267g0010	Probable ubiquitin‐conjugating enzyme E2 23	MA_10437267	Medium	1	1	RDA‐raw/GF‐raw
MA_112985g0010	Ribosomal lysine N‐methyltransferase 3‐like isoform X1	MA_112985	Medium	1	1	RDA‐raw/GF‐raw
MA_115260g0010	Uncharacterized protein LOC18422833	MA_115260	Medium	1	1	RDA‐raw/GF‐raw
MA_118687g0010	5‐formyltetrahydrofolate cycloligase	MA_118687	Medium	1	1	RDA‐raw/GF‐raw
MA_118899g0010	Probable phosphatase 2C 59 isoform X1	MA_118899	Medium	1	1	RDA‐raw/GF‐raw
MA_119384g0010	Pyruvate dehydrogenase (acetyl‐transferring) mitochondrial	MA_119384	Medium	1	1	RDA‐raw/GF‐raw
MA_141850g0010	H ACA ribonucleo complex subunit 2	MA_141850	Medium	1	1	RDA‐raw/GF‐raw
MA_15092g0010	Unknown	MA_15092	Medium	1	1	RDA‐raw/GF‐X
MA_340419g0010	na	MA_340419	Medium	1	1	RDA‐raw/GF‐raw
MA_361488g0010	Chloroplastic	MA_361488	Medium	1	1	RDA‐corr/GF‐X
MA_477513g0010	Thioredoxin chloroplastic‐like	MA_477513	Medium	1	1	RDA‐raw/GF‐raw
MA_486420g0010	TITAN isoform X1	MA_486420	Medium	1	2	RDA‐raw/GF‐raw
MA_50056g0010	GRIP	MA_50056	Medium	1	1	GF‐raw/GF‐X
MA_66499g0010	Pentatricopeptide repeat‐containing At2g13600‐like	MA_66499	Medium	1	1	RDA‐raw/GF‐X
MA_705952g0010	Adenylylsulfatase HINT3 isoform X2	MA_705952	Medium	1	1	RDA‐raw/GF‐raw
MA_737390g0010	Unknown	MA_737390	Medium	1	1	RDA‐raw/GF‐raw
MA_780508g0010	na	MA_780508	Medium	1	1	RDA‐raw/GF‐raw
MA_82878g0010	na	MA_82878	Medium	1	1	RDA‐raw/GF‐raw
MA_86597g0020	Nucleolar pre‐ribosomal‐associated 1	MA_86597	Medium	1	1	RDA‐raw/GF‐raw
MA_9232936g0010	Zinc finger 593	MA_9232936	Medium	1	1	RDA‐raw/GF‐raw
MA_9293176g0010	Translation elongation factor‐1	MA_9293176	Medium	1	1	RDA‐raw/GF‐raw
MA_93738g0010	UDP glucose: glyco glucosyltransferase	MA_93738	Medium	1	1	RDA‐raw/GF‐raw
MA_95297g0010	DNA topoisomerase 6 subunit B	MA_95297	Medium	1	1	RDA‐raw/GF‐raw
MA_9859326g0010	Unknown	MA_9859326	Medium	1	1	RDA‐raw/GF‐raw
MA_102606g0010	CHROMATIN REMODELING 4‐like isoform X1	MA_102606	High	2	1	RDA‐raw/GF‐X
MA_10427262g0010	Accelerated cell death 11	MA_10427262	High	2	1	RDA‐raw/GF‐X
MA_10428623g0010	High mobility group B 15 isoform X1	MA_10428623	High	2	1	RDA‐raw/GF‐X
MA_10436445g0020	TPR2 isoform X1	MA_10436445	High	2	1	RDA‐raw/GF‐X
MA_104862g0010	Duplicated homeodomain‐like superfamily isoform 1	MA_104862	High	2	2	RDA‐raw/GF‐X
MA_123124g0020	Apoptotic chromatin condensation inducer in the nucleus	MA_123124	High	2	1	RDA‐raw/RDA‐corr
MA_125412g0010	Probable L‐type lectin‐domain containing receptor kinase	MA_125412	High	2	1	RDA‐raw/GF‐X
MA_41156g0010	DNA damage‐binding 1	MA_41156	High	2	1	RDA‐raw/GF‐X
MA_474261g0010	Pentatricopeptide repeat‐containing chloroplastic‐like	MA_474261	High	2	1	RDA‐raw/GF‐X
MA_49114g0010	na	MA_49114	High	2	1	RDA‐raw/GF‐X
MA_66837g0010	MARD1	MA_66837	High	2	1	RDA‐raw/RDA‐corr
MA_767345g0010	B‐cell receptor‐associated	MA_767345	High	2	1	RDA‐raw/RDA‐corr
MA_86450g0010	Glutathione S‐transferase DHAR2‐like	MA_86450	High	2	1	RDA‐raw/RDA‐corr/GF‐X
MA_91467g0010	Pectate lyase	MA_91467	High	2	1	RDA‐raw/GF‐X
MA_9156578g0010	Serine threonine‐ kinase pakA‐like	MA_9156578	High	2	1	RDA‐raw/GF‐X
MA_10071760g0010	Beta‐galactosidase 8	MA_10071760	Low	2	1	RDA‐raw/GF‐X
MA_10433070g0010	na	MA_10433070	Medium	2	1	RDA‐raw/RDA‐corr
MA_10433219g0010	D‐tagatose‐1,6‐bisphosphate aldolase subunit kbaZ	MA_10433219	Medium	2	1	RDA‐raw/RDA‐corr
MA_300643g0010	Probable LRR receptor‐like serine threonine‐ kinase At1g56140	MA_300643	Medium	2	2	RDA‐raw/RDA‐corr
MA_72912g0010	na	MA_72912	Medium	2	2	RDA‐raw/RDA‐corr
MA_78838g0010	rRNA methylase	MA_78838	Medium	2	1	RDA‐raw/GF‐X
MA_97492g0010	Anaphase‐promoting complex subunit 2	MA_97492	Medium	2	1	RDA‐raw/GF‐X

na, not available.

The GO functional enrichment test returned one enriched biological process (false discovery rate (FDR) < 0.05): carbohydrate metabolic process (GO: 0005975), and three enriched molecular functions: protein binding (GO: 0005515; and its parent GO‐term, binding (GO: 0005488)), and polygalacturonate 4‐alpha‐galacturonosyltransferase activity (GO: 0047262).

### Distribution of adaptive alleles across climatic and geographic landscapes

**Fig. 5:**
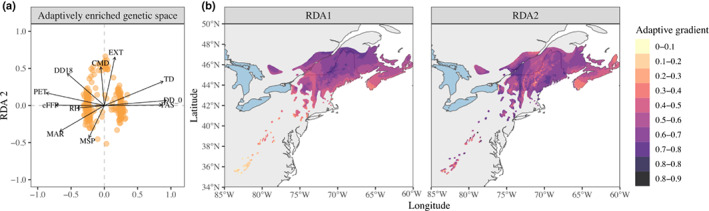
Red spruce adaptive landscape with (a) a redundancy analysis (RDA) biplot showing the association between adaptive loci and climatic drivers of adaptation in the adaptively enriched genetic space and (b) spatial projection of adaptive genetic turnover through extrapolation of the RDA model to the complete range of red spruce. The loci coordinates used in (a) have been multiplied by four for better visibility and the scale of (b) shows gradual variation between the two extreme genetic compositions (0 and 1) associated with extreme values of each RDA axis.

The adaptively enriched RDA conducted on the 240 putatively adaptive loci identified two main gradients of adaptation to climate in red spruce (Fig. 5). The first axis of variation (RDA1, 94.1% of variance) contrasted localities characterized by a large amount of precipitation as snow (PAS), many degree‐days below 0°C (DD_0) and high continentality (TD) – typically found in Southern Quebec and Northern USA mountainous areas – with localities experiencing higher potential evapotranspiration (PET), a later end of the frost‐free period (eFFP) and a higher degree of solar radiation (MAR) – found primarily in the Central and Southern Appalachians. The second axis, RDA2 (2.6% of variance) was more strongly associated with an altitudinal gradient, differentiating lowland areas with higher degree‐days above 18°C (DD18), higher extreme temperature events (EXT) and higher climatic moisture deficit (CMD) from high elevation locations, which experience more summer precipitation (MSP) and lower temperatures.

**Fig. 6:**
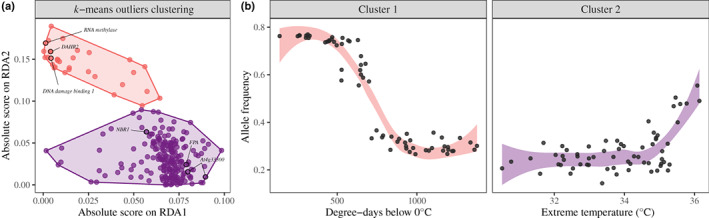
Adaptive loci clustering and clines along climatic gradients. (a) Results of the *k*‐mean clustering procedure performed on the absolute value of the outlier loci loadings along the two first axes of the adaptively enriched redundancy analysis (RDA). (b) Adaptive genetic clines along gradients of growing‐degree days below 0°C and annual mean precipitation gradients, with their 95% confidence intervals represented by colored bands around the fitted line.

We also found that the two main gradients of adaptation were driven by two different clusters among the identified 240 putatively adaptive loci, which were grouped into ‘modules’ based on their RDA loadings (Fig. 6). The first module (Cluster 1) gathered most of the adaptive loci (207/240) and included, among others, the genes *At4g33300*, *NBR1*, *HSFB‐2B* and *FPA*, which are known to be involved in adaptation along temperature and drought gradients (Fig. 6a). The mean population allele frequency within Cluster 1 returned a strongly clinal pattern of co‐variation with RDA1, especially with degree‐days below 0°C (Figs 6b, S6). The second module (Cluster 2) consisted of 33 loci that included genes involved in response to acute stresses such as an rRNA methylase, *DNA damage‐binding 1*, and *DHAR2* (Fig. 6a). Mean allele frequencies for this cluster covaried with RDA2, which was strongly correlated with extreme maximum temperature (Figs 6b, S6).

## Discussion

### Red spruce shows local adaptation to climate across its range

We detected in red spruce common gardens a strong negative correlation between seedling growth and climate transfer distance and estimated that around a third of the variation in height growth could be attributed to disruption of local adaptation caused by moving seedlings from their source climates. This negative impact of climate transfer on seedling performance, assessed for many localities (65) and at three garden sites, provides strong confirmation that red spruce is genetically adapted to local climatic conditions. Our results confirm that red spruce is no exception to the ubiquity of climate adaptation among members of the *Picea* genus (Mimura & Aitken, 2010; Rossi, 2015; Milesi *et al*., 2019; Depardieu *et al*., 2020).

At all three common garden sites, we found that the reduction in seedling performance could be substantial, with up to 10 cm of reduction in height over two growing seasons, representing a 25% height increment decrease in comparison to the maximal value observed (*c*. 40 cm). We used height growth here as a proxy of early‐life fitness to demonstrate local adaptation (Blanquart *et al*., 2013), and other work by our group indicates that genetic divergence in adaptive phenology traits (e.g. bud break and bud set) likely underlie these growth differences (Prakash *et al*., 2022). Another recent study of red spruce provenances planted at five different trial sites in eastern Canada found a similar result in adult trees, showing that two fitness traits (height and diameter at breast height (DBH)) were correlated with climate transfer distance (Li *et al*., 2020). Li *et al*. (2020) employed garden sites that had cooler temperature regimes in comparison with most of their source locations, and observed a negative fitness impact of transfer to colder climates, with a strong influence of mean annual temperature, length of the frost‐free period and number of growing degree days above 5°C. All together, these results confirm that red spruce exhibits local adaptation to climate and provides rationale for identifying environmental gradients and genomic variation that underlie this adaptation.

### Collinearity between geography, neutral genetic structure, and climatic gradients

Characterizing genetic–environment relationships remains a source of intense methodological and empirical development (Savolainen *et al*., 2013; Ćalić *et al*., 2016; Hoban *et al*., 2016) and there is growing appreciation of the relative contribution of climatic selection, geographic distance and demographic history to spatial variation in allele frequencies (Joost *et al*., 2013; Orsini *et al*., 2013; Wang & Bradburd, 2014). However, a key challenge is that these factors tend to be confounded in natural landscapes (Forester *et al*., 2018; Price *et al*., 2019). For red spruce, we found that a large portion (45%) of the explained variance in locality allele frequencies was shared between these three factors, likely reflecting south‐to‐north post‐glacial expansion along the Appalachian Mountains, which created collinearity between red spruce's geographic distribution, genetic structure, and climatic gradients (T. Capblancq *et al*., unpublished). This latitudinal alignment presents a challenge for genome scans attempting to disentangle the variation resulting from neutral vs selective processes. It has become common practice to condition genome scans with proxies of neutral population structure, which reduces the risk of false positives (De Villemereuil *et al*., 2014; Frichot *et al*., 2015). However, removing the effect of population structure also reduces or eliminates signals of climatic selection (Savolainen *et al*., 2013), thereby increasing false negatives, i.e. failing to discover genes actually involved in climate adaptation (Bergelson & Roux, 2010; Anderson *et al*., 2011). When we corrected our GEA scans by neutral population structure, our results showed that (1) the overall correlation between climate and genetic variation dramatically shrank; (2) adaptive loci identified by both RDA and GF substantially decreased; and (3) we failed to find many candidate genes associated with response to abiotic and biotic stress in other plant species that were only detectable in red spruce when not accounting for population structure. This failure to detect a strong signal of climate adaptation after correcting for population structure is likely the result of missing many or most of the loci contributing to climate adaptation, evidence for the existence of which comes from phenotypic measurements in our common garden experiments (Fig. 2). Further, we found that among RDA models fitted on different sets of outliers, climate consistently explained more of the allele frequency variance (*R* ^2^) for sets of outliers that were not corrected for population structure, and more than twice the *R* ^2^ of random loci (Table S2). This suggests that a large amount of the genetic architecture underlying climate adaptation in red spruce is only captured in the uncorrected genome scans, even if the latter also come with a high likelihood of false positive loci due to neutral population structure.

While our findings may represent an extreme example of confounding between neutral and adaptive genetic variation, we suspect this issue is not unique to red spruce. Other temperate species experienced south–north expansions after the LGM (Hewitt, 1999) and many adaptations are associated with photoperiod and/or temperature, which are themselves correlated with latitudinal gradients, setting up the pre‐conditions for confounding geography, genetic structure, and selection. As such, we believe that correcting GEA for population structure might be counterproductive in some situations where environmental variation strongly covaries with recolonization routes. If we want to capture genomic regions involved in adaptation along these gradients, we may have to accept a higher frequency of false positives as well and use additional sources of evidence such as functional annotation or experimental validation to help refine our understanding of the role that individual genes or genomic variants may play. This is an important issue confronting the field of ecological genomics, and our study serves as one case that attempts to address this challenge.

### Candidate genes of adaptation to climate in red spruce

Three main interconnected axes of adaptation to climate are usually described in the literature of temperate and boreal coniferous species: drought tolerance (Moran *et al*., 2017), cold hardiness (Aitken *et al*., 2004; Chang *et al*., 2021) and phenological timing of growth and dormancy (Gyllenstrand *et al*., 2007). The set of putatively adaptive genes we identified in red spruce using different GEA methods includes genes involved in all three of these adaptive pathways.

Degree days below 0°C (DD_0) and extreme temperature (EXT) were among the most important climatic factors in the genome scans and we found many genes involved in response to heat and/or drought stress, confirming the role of both extreme cold and heat stress in driving climate adaptation in spruce (Depardieu *et al*., 2020, 2021). Additionally, we observed significant functional enrichment for carbohydrate metabolism, which is described in the literature as an important component of drought response in conifers (Moran *et al*., 2017), possibly because the accumulation of sugars (e.g. carbohydrates) help plants to resist dehydration and desiccation (Perdiguero *et al*., 2012). We also identified some genes of the ABA (abscisic acid) pathway (e.g. *MARD1*). ABA is known to initiate responses to drought stress (Hamanishi & Campbell, 2011), with high ABA concentration acting as a signal for plants to close their stomata and prevent water loss (Moran *et al*., 2017). We also found genes associated with other aspects of drought tolerance, such as the *NBR1* involved in heat and drought stress (Zhou *et al*., 2013), *HSFB‐2B* involved in heat stress regulation (Guo *et al*., 2016), and *ACD11* (accelerated cell death 11), whose overexpression is triggered by an increase of ABA and improves salt and drought tolerance in *Arabidopsis thaliana* (Li, 2019).

Multiple studies also show the importance of circadian clock genes in tree adaptation to phenology cues such as temperature and photoperiod (Holliday *et al*., 2010; Chen *et al*., 2012; Keller *et al*., 2012; Olson *et al*., 2013). According to the results of the different GEA procedures we used in this study, the *FPA* gene was one of the strongest selection candidates, which regulates flowering time in *Arabidopsis thaliana* via the autonomous pathway, independent of daylength (Schomburg *et al*., 2001). In common garden studies by our group, we have shown strong genetic differentiation in bud set phenology traits in red spruce, including at broad latitudinal scales (Prakash *et al*., 2022) and at fine scales between genotypes sourced from low vs high elevations on the same mountain that experience essentially identical photoperiod regimes (Butnor *et al*., 2019; Verrico, 2021). This could suggest that red spruce phenology is driven by temperature in addition to, or in interaction with, photoperiod, consistent with results from growth chamber experiments in white spruce (Hamilton *et al*., 2016). *FPA* has also been shown to play a role in post‐transcriptional modification of mRNAs from other expressed genes in response to dehydration stress (Sun *et al*., 2017), making this candidate a particularly intriguing target for further investigation.

Lastly, genes involved in pathogen resistance are present in our list of candidates, as has been reported in other GEA studies of climate selection in forest trees (Chhatre *et al*., 2019). Pathogen resistance is a well‐known driver of local adaptation in trees (Fetter *et al*., 2017), and pathogen prevalence or resistance often covaries along latitudinal and temperature gradients (Moreira *et al*., 2014). In a recent genome‐wide association study (GWAS) in Norway spruce, pathogen resistance was found to be associated with a regulator of the ABA pathway, which is also involved in plant water balance, suggesting the potential for pleiotropy between biotic and abiotic stress (Capador‐Barreto *et al*., 2021). Along the same lines, one of our top candidate genes (*At4g33300*) is involved in drought tolerance but also known to be associated with pathogen defense response in silver fir (Behringer *et al*., 2015).

Future work is needed to better understand how these candidate genes underlie variation in climate‐adaptive traits. Nonetheless it is already clear that these loci are acting through pathways that contribute to variation in early‐life seedling performance in our three common gardens. Indeed, the association of individual loci identified as outliers by our GEA procedure explained around 1–3% of Height Growth on average, far exceeding the association of nonoutliers. This level of variance explained by single loci is consistent with the highly polygenic nature of quantitative traits involved in local adaptation in plants (Savolainen *et al*., 2013; Lee *et al*., 2014; Josephs *et al*., 2017).

### Adaptive genetic variation across climatic landscapes

Interestingly, we found that two distinct groups of genes were associated with two different environmental gradients and showed different patterns of variation across the landscape. The first axis of adaptive variation, driven by a majority of the identified candidate genes (103/125), showed clinal variation in allele frequencies along a gradient defined by the number of degree‐days below 0°C. Across the 207 SNPs associated with these 103 genes, average allele frequencies varied from 0.25 to 0.77 and some specific loci showed even larger differential in allele frequency (e.g. the *FPA* gene, Fig. S6). The inflection point of the genetic cline was found around 700 degree‐days below 0°C, suggesting a coordinated shift at this portion of the gradient from one set of alleles to the other across multiple genes. This pattern also suggests the genetic basis of local adaptation in these genes may reflect antagonistic pleiotropy (Anderson *et al*., 2011; Savolainen *et al*., 2013), with one allele being better suited for northern and continental regions – characterized by many below freezing days, abundant snow, low potential evapotranspiration, and short growing seasons – whereas the alternative allele is beneficial at lower latitudes and more coastal areas – characterized by longer growing seasons. This cluster included many genes involved in response to abiotic stress, especially heat and drought (e.g. *NBR1*), but also genes involved in phenology or response to stress (e.g. *FPA*) and resistance to pathogens (e.g. *At4g33300*).

The second axis of adaptation was supported by fewer genes (22) and showed a different pattern of variation. Here, the mean allele frequency across the 33 associated SNPs remained low along the associated climatic gradient (e.g. extreme temperature/elevation) before increasing substantially when approaching the upper bound of the gradient, suggesting a threshold effect. Among the 22 genes included in this group, we found genes involved in response to acute stresses in other plant species, for example *DAHR2*, which plays a role in adaptation to high‐light conditions in *Arabidopsis thaliana* (Terai *et al*., 2020) or *DNA damage‐binding 1*, involved in *Brassicaceae* adaptation to high altitude (Guo *et al*., 2018). This suggests that specific alleles are selected at low elevations where red spruce trees more often experience extreme events of heat and/or drought but are not under selection in areas where climatic conditions never reach such extremes. Experimental testing would be required to confirm this hypothesis, but it could be evidence of conditional neutrality where some alleles are favored in one environment and are neutral in other environments (Anderson *et al*., 2011).

### Conclusion

Our results unravel the pattern of local adaptation to climate in red spruce in the face of confounding effects of demographic history. Local adaptation is reflected in divergent adaptive gene pools along a latitudinal gradient but also at specific lowland localities. We showed that seedling fitness decreased due to local maladaptation induced by a climate‐transfer experiment into three common gardens. This raises the question of potential future disruption of existing gene–environment relationships by rapid climate change, which could eventually lead to population decline. We also showed that genes associated with tolerance to drought, cold hardiness and phenology were important components of red spruce adaptation to climate. For these genes, we found large allelic turnover along climatic gradients, confirming the presence of standing genetic variation that could be used to avoid maladaptation. These results can now be used to develop models and tools that can take advantage of information on adaptive intraspecific variation to help inform future management strategies and buffer the negative impact of climate change on forest ecosystems.

## Author contributions

SRK and MCF conceived the study and collected the samples. TC analyzed genomic data and conducted the statistical analyzes with help from SL, SRK and MCF. SL analyzed the climate data and helped with the estimation of climate transfer distances. TC wrote a first draft of the manuscript and all authors provided critical feedbacks.

## Supporting information


**Fig. S1** Principal component analysis of the climatic conditions experienced by red spruce.
**Fig. S2** Influence of climate transfer distances on seedling fitness represented by mean height growth per locality at the three common garden sites.
**Fig. S3** Correlogram of the different variables used to explain the genetic variance during the variance partitioning procedure.
**Fig. S4** Manhattan plots showing the results of the four different genome scans (RDA, redundancy analysis; GF, gradient forest).
**Fig. S5** Venn diagrams showing the overlap between the top 0.2% loci resulting from multivariate (RDA, GF) and univariate (lfmm, bayenv2) genome scans.
**Fig. S6** Variation of allele frequency for six important genes involved in red spruce adaptation to local climates.
**Notes S1** Web literature search of the genes identified as potentially involved in red spruce adaptation to climate.
**Table S1** Table summarizing information about sampled localities and families.
**Table S2** Table showing the result of redundancy analysis models regressing population allele frequencies of different sets of loci against the 11 selected climate variables.
**Table S3** Summary of genetic variants and their functional annotation identified by mapping the exome capture sequences against the Norway spruce annotated genome.
Please note: Wiley Blackwell are not responsible for the content or functionality of any Supporting Information supplied by the authors. Any queries (other than missing material) should be directed to the *New Phytologist* Central Office.

## Data Availability

Sequence data are available in the form of fastq files with the raw reads of each individual in SRA NCBI database (https://www.ncbi.nlm.nih.gov/sra/), deposited as PRJNA625557 bioproject. Trait data and genotype dosages for the 917 234 SNPs and 332 individuals genotyped are available on the Dryad repository (https://doi.org/10.5061/dryad.z08kprrgm).
