# Supplementary material for: From common gardens to candidate genes: exploring local adaptation to climate in red spruce
Source: New Phytol. 2022 Oct 13;237(5):1590–605. doi: 10.1111/nph.18465 (PMC10092705; doi:10.1111/nph.18465)
Supplement: Supplementary file 1 — Fig. S1 Principal component analysis of the climatic conditions experienced by red spruce. Fig. S2 Influence of climate transfer distances on seedling fitness represented by mean height growth per locality at the three common garden sites. Fig. S3 Correlogram of the different variables used to explain the genetic variance during the variance partitioning procedure. Fig. S4 Manhattan plots showing the results of the four different genome scans (RDA, redundancy analysis; GF, gradient forest). Fig. S5 Venn diagrams showing the overlap between the top 0.2% loci resulting from multivariate (RDA, GF) and univariate (lfmm, bayenv2) genome scans. Fig. S6 Variation of allele frequency for six important genes involved in red spruce adaptation to local climates. Notes S1 Web literature search of the genes identified as potentially involved in red spruce adaptation to climate. Table S1 Table summarizing information about sampled localities and families. Table S2 Table showing the result of redundancy analysis models regressing population allele frequencies of different sets of loci against the 11 selected climate variables. Table S3 Summary of genetic variants and their functional annotation identified by mapping the exome capture sequences against the Norway spruce annotated genome. Please note: Wiley Blackwell are not responsible for the content or functionality of any Supporting Information supplied by the authors. Any queries (other than missing material) should be directed to the New Phytologist Central Office. [file NPH-237-1590-s001.pdf]

New Phytologist **Supporting Information**

From common gardens to candidate genes: exploring local adaptation to climate in red spruce

Thibaut CAPBLANCQ, Susanne LACHMUTH, Matthew C. FITZPATRICK and Stephen R. KELLER

Article acceptance date: 9 August 2022

The following Supporting Information is available for this article:

Supporting Information Figure S1: Principal component analysis of the climatic conditions experienced by red spruce.

Supporting Information Figure S2: Influence of climate transfer distances on seedling fitness represented by mean height growth per locality at the three common garden sites.

Supporting Information Figure S3: Correlogram of the different variables used to explain the genetic variance during the variance partitioning procedure.

Supporting Information Figure S4: Manhattan plots showing the results of the four different genome scans (RDA, redundancy analysis; GF, gradient forest).

Supporting Information Figure S5: Venn diagrams showing the overlap between the top 0.2% loci resulting from multivariate (RDA, GF) and univariate (LFMM, Bayenv2) genome scans.

Supporting Information Figure S6: Variation of allele frequency for six important genes involved in red spruce adaptation to local climates.

Supporting Information Table S1: Table summarizing information about sampled localities and families.

Supporting Information Table S2: Table showing the result of redundancy analysis (RDA) models regressing population allele frequencies of different sets of loci against the 11 selected climate variables.

Supporting Information Table S3: Summary of genetic variants and their functional annotation identified by mapping the exome capture sequences against the Norway spruce annotated genome.

Supporting Information Notes S1: Web literature search of the genes identified as potentially involved in red spruce adaptation to climate.

Fig. S1: Principal component analysis of the climatic conditions experienced by red spruce. The left panel shows the extent of climatic occupancy of the sampled source localities (orange) across the complete climatic niche of red spruce (green + orange). The right panel shows the importance of the different climatic variables in discriminating red spruce forests' environments.

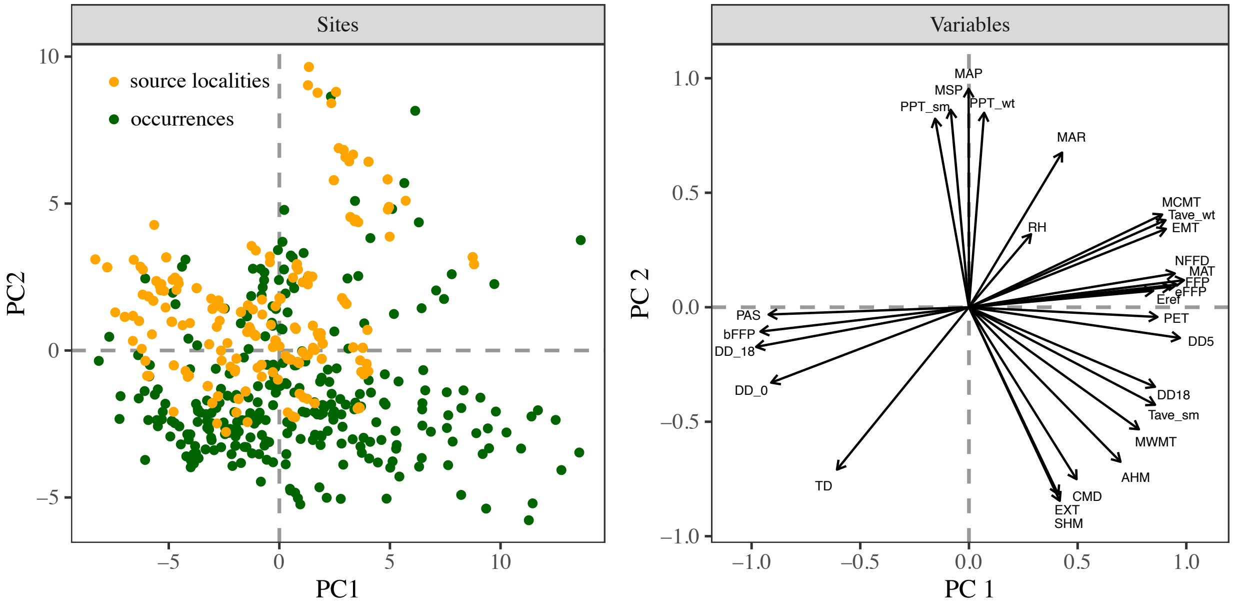

Fig. S2: Association between climate transfer distance based on 11 variables and seedling fitness represented by mean height growth per source locality at the three red spruce common garden sites. The significance of the association was tested in each garden using a linear regression, the resulting p-values and R^2 are shown on each panel. Lines show model predictions and the gray areas show the 95% confidence intervals.

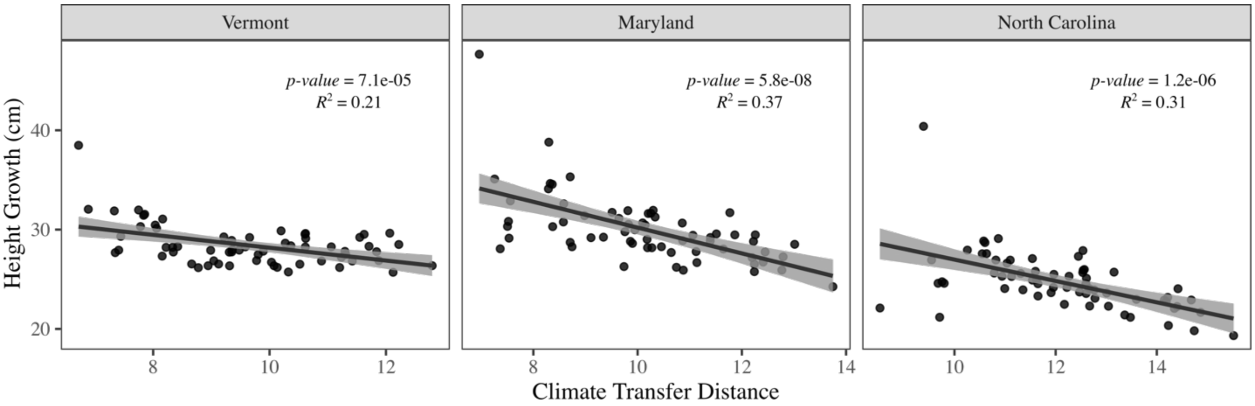

Fig. S3: Correlogram of the different variables used to explain the genetic variance of red spruce during the variance partitioning procedure. The color scale is based on the correlation coefficient estimated between each pair of variables and gives an idea of the strength and direction of the pairwise associations.

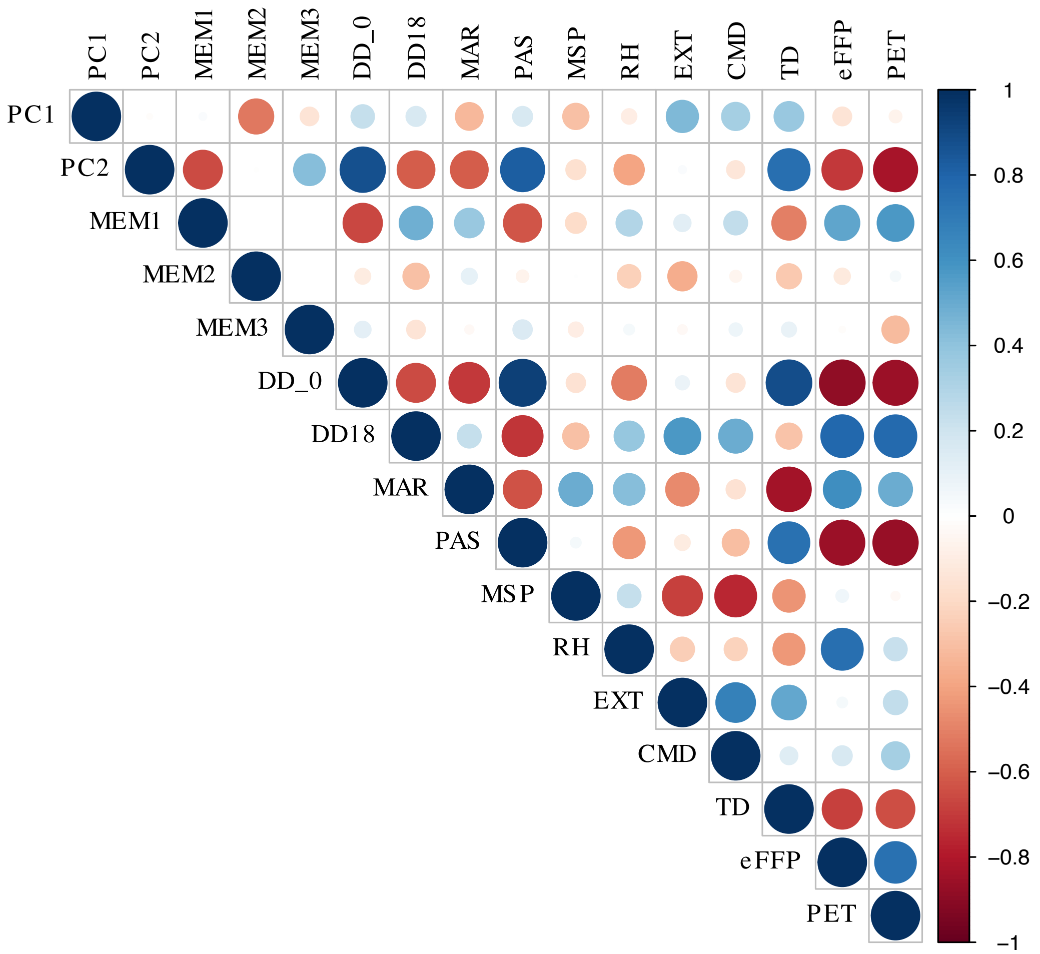

Fig. S4: Manhattan plots showing the results of the four different genome scans conducted on the red spruce dataset (RDA, redundancy analysis; GF, gradient forest).

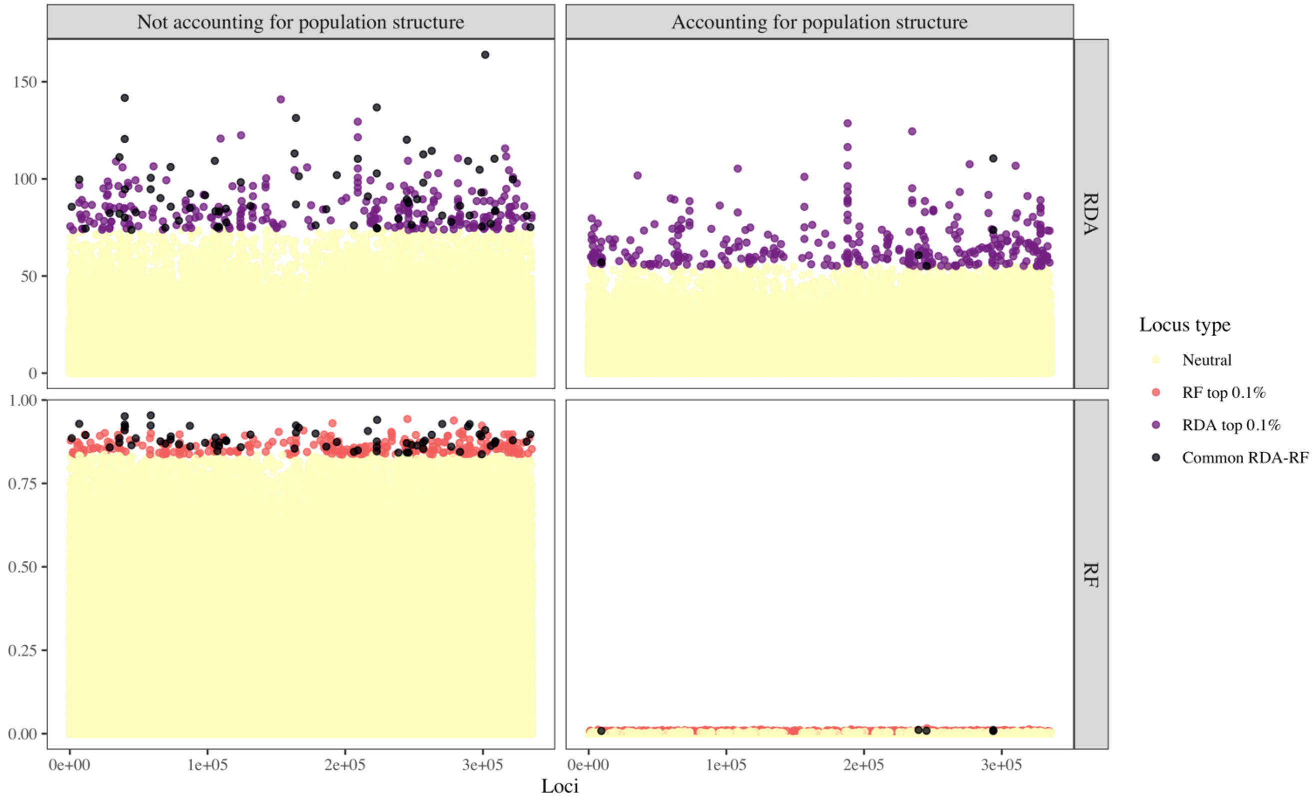

Fig. S5: Venn diagrams showing the overlap between the top 0.2% loci resulting from multivariate (RDA, GF) and univariate (LFMM, Bayenv2) genome scans when (a) population structure was corrected and (b) running RDA and GF on raw allele frequencies. Important note: the number of loci is higher for LFMM and Bayenv2 because it includes the top 0.2% loci associated with each of the 11 environmental variables.

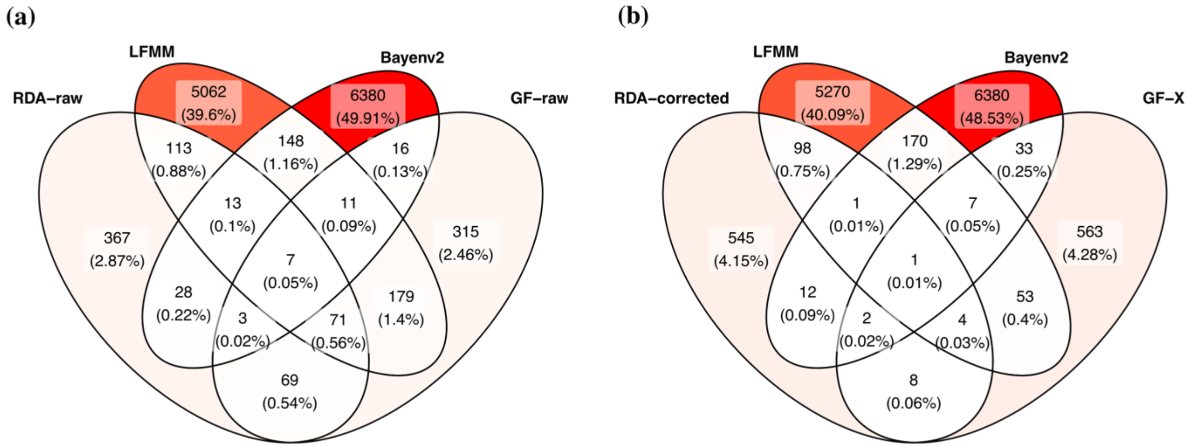

Fig. S6: Variation of allele frequency for six important genes involved in red spruce adaptation to local climates. The genes of the top row belong to Cluster 1 whereas the genes of the bottom row belong to Cluster 2.

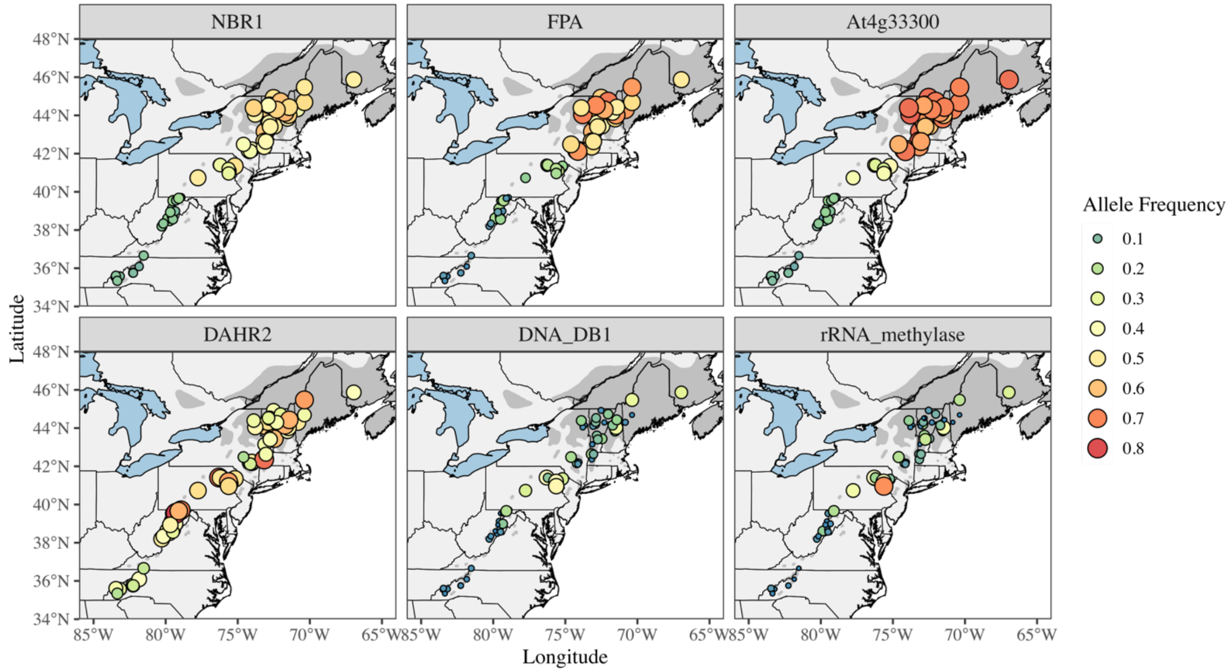

Table S1: Table summarizing information about sampled localities and families.

Family	Site	Tree	State	Region	Latitude	Longitude	Elevation
AB_05	AB	5	TN	E	35.55297	83.49438	1812
AB_08	AB	8	TN	E	35.55212	83.49259	1785
AB_12	AB	12	TN	E	35.5389	83.49463	1750
AB_16	AB	16	TN	E	35.53882	83.49534	1738
AB_18	AB	18	TN	E	35.53945	83.49375	1769
ALB_01	ALB	1	ME	C	44.3067	70.84019	455
ALB_02	ALB	2	ME	C	44.30564	70.84081	524
ALB_03	ALB	3	ME	C	44.30359	70.84096	523
ALB_05	ALB	5	ME	C	44.30182	70.83729	573
ALB_06	ALB	6	ME	C	44.30061	70.83641	589
APP_01	APP	1	VT	C	44.20933	72.9295	743
APP_02	APP	2	VT	C	44.21172	72.92999	780
APP_03	APP	3	VT	C	44.22097	72.92639	896
APP_04	APP	4	VT	C	44.2114	72.93122	741
APP_05	APP	5	VT	C	44.21034	72.93051	733
APP_06	APP	6	VT	C	44.21235	72.93502	658
ASC_01	ASC	1	VT	C	43.43787	72.45218	883
ASC_02	ASC	2	VT	C	43.43983	72.45328	847
ASC_03	ASC	3	VT	C	43.44192	72.45589	861
ASC_04	ASC	4	VT	C	43.4426	72.45713	870
ASC_05	ASC	5	VT	C	43.44376	72.45867	879
ASC_06	ASC	6	VT	C	43.44379	72.45434	967
B_02	B	2	VT	C	44.10929	73.0714	311
B_03	B	3	VT	C	44.10897	73.0712	325
B_04	B	4	VT	C	44.10908	73.0713	328
B_05	B	5	VT	C	44.10836	73.0715	310
B_06	B	6	VT	C	44.10889	73.0714	321
BAL_01	BAL	1	NY	C	44.1528	73.64591	570
BAL_02	BAL	2	NY	C	44.1517	73.65127	620
BAL_03	BAL	3	NY	C	44.15337	73.65371	650
BAL_04	BAL	4	NY	C	44.15409	73.65688	670
BAL_05	BAL	5	NY	C	44.15415	73.65799	698
BAL_10	BAL	10	NY	C	44.16008	73.66681	918
BBE_01	BBE	1	NH	C	43.83596	71.55484	365
BBE_02	BBE	2	NH	C	43.83457	71.54906	368
BBE_03	BBE	3	NH	C	43.83484	71.53966	373
BBE_04	BBE	4	NH	C	43.83367	71.53963	383
BBE_05	BBE	5	NH	C	43.83176	71.54305	374
BER_01	BER	1	ME	C	44.69079	70.37595	481
BER_02	BER	2	ME	C	44.69043	70.37637	487
BER_03	BER	3	ME	C	44.68987	70.37686	482
BER_04	BER	4	ME	C	44.68953	70.37609	487
BER_05	BER	5	ME	C	44.68899	70.37624	492

Table S2: Table showing the result of redundancy analysis (RDA) models regressing population allele frequencies of different sets of loci against the 11 selected climate variables. The models were run for each of the four Genotype-Environment Association (GEA) scans top hit loci sets, for the set of 109 putatively adaptive loci (loci common to two different GEAs) and a set of random loci. The random set consisted of 336 loci (equivalent to the GEA sets) randomly selected among the loci that were not selected by any of the four multivariate GEAs.

**RDA models**	**Df**	**Variance**	**F**	$\mathbf{R^2}$	**p (> F)**
$Freq_{random loci}$ ~ climatic variables	11	1.7	2.10	0.31	*0.001* ***
$Freq_{RDA-raw}$ ~ climatic variables	11	19.4	11.84	0.71	*0.001* ***
$Freq_{RDA-corrected}$ ~ climatic variables	11	8.0	3.63	0.43	*0.001* ***
$Freq_{GF-raw}$ ~ climatic variables	11	18.6	25.45	0.84	*0.001* ***
$Freq_{GF-X}$ ~ climatic variables	11	9.3	7.84	0.62	*0.001* ***
$Freq_{common}$ ~ climatic variables	11	9.5	19.72	0.81	*0.001* ***

Table S3: Summary of genetic variants and their functional annotation identified by mapping the exome capture sequences against the Norway spruce annotated genome.

Category	Frequency
Downstream	32 (13.3%)
Nonsynonymous	14 (5.8%)
Synonymous	34 (14.2%)
Upstream	34 (14.2%)
Intron	33 (13.8 %)
Intergenic	93 (38.8 %)
Total	240 (100%)

Notes S1: This supplementary material lists all the candidate genes identified with the different genome scan analyses (see Table 2). We searched the literature for involvement of these genes in adaptation to climate in other plant species. Our findings are listed below for the two different cluster of genes identified (see Figure 6).

CLUSTER 1

- At4g33300 protein, involved in defense response and drought resistance in *Abies alba* (Behringer *et al.*, 2015) and *Arabidopsis thaliana* (Bonardi *et al.*, 2017)
- NBR1, involved in heat and drought stress in *Arabidopsis thalinana* (Zhou *et al.*, 2013)
- HSFB-2B, commonly found involved in heat stress regulation in plants (Guo *et al.*, 2016)
- FPA, regulates flowering time in *Arabidopsis thaliana* via a pathway that is independent to daylight (Schomburg *et al.*, 2001)
- CML49, calcium-binding proteins regulating plant growth and development, as well as in the resistance mechanisms to various biotic and abiotic stresses (Shi & Du, 2020)
- cysteine and histidine-rich domain-containing RAR1, contributes to disease resistance in *Arabidopsis thalinana* (Tornero *et al.*, 2002)
- VIN3 1, involved in both the vernalization and photoperiod pathways by regulating expression of related floral repressors in *Arabidopsis thalinana* (Sung *et al.*, 2006)
- probable carotenoid cleavage dioxygenase chloroplastic*, nothing found linked to plant adaptation to climate.*
- rac-like GTP-binding 5*, nothing found linked to plant adaptation to climate.*
- beta-galactosidase 5-like*,* beta-galactosidases are associated with cold tolerance in citrus (Lang *et al.*, 2005).
- ubiquitin carboxyl-terminal hydrolase 18-like*, nothing found linked to plant adaptation to climate.*
- pentatricopeptide repeat-containing At4g14850*, nothing found linked to plant adaptation to climate.*
- polyadenylate-binding RBP47B, plays a role in the formation of stress granules, which are used to selectively store mRNA for use in response to a stress such as heat (Kosmacz *et al.*, 2018)
- E3 ubiquitin ligase listerin, large family of proteins whose members are involved in regulation of a number of biological processes including light response, biotic and abiotic stress tolerance and DNA repair (Mazzucotelli *et al.*, 2006).

- GPDH: glycerol-3-phosphate dehydrogenase [NAD(+)] cytosolic-like, response to various abiotic stress in different species and especially osmotic stress in maize (Zhao *et al.*, 2018).
- DCAF8: DDB1- and CUL4-associated factor 8*, nothing found linked to plant adaptation to climate.*
- ZFN2: zinc finger CCCH domain-containing ZFN-like isoform X2*, nothing found linked to plant adaptation to climate.*
- probable cyclic nucleotide-gated ion channel chloroplastic*, nothing found linked to plant adaptation to climate.*
- RETICULATA-RELATED chloroplastic-like*, nothing found linked to plant adaptation to climate.*
- probable galacturonosyltransferase 13 isoform X1*, nothing found linked to plant adaptation to climate.*
- importin subunit beta-1, plays a role in the ABA response and drought tolerance (Luo *et al.* 2013)
- myb-related Zm38-like*, nothing found linked to plant adaptation to climate.*
- probable galacturonosyltransferase-like 3*, nothing found linked to plant adaptation to climate.*
- probable ubiquitin-conjugating enzyme E2 18*, nothing found linked to plant adaptation to climate.*
- TRANSPORT INHIBITOR RESPONSE 1-like*, nothing found linked to plant adaptation to climate.*
- rare cold inducible, small hydrophobic membrane proteins induced by salt, cold, and drought stresses in many plant species (Kim *et al.*, 2021)
- serine--glyoxylate aminotransferase*, nothing found linked to plant adaptation to climate.*
- aspartate carbamoyltransferase chloroplastic*, nothing found linked to plant adaptation to climate.*
- pentatricopeptide repeat-containing At2g13600-like*, nothing found linked to plant adaptation to climate.*
- plastid division PDV1*, nothing found linked to plant adaptation to climate.*
- DUF179 domain-containing*, nothing found linked to plant adaptation to climate.*
- coatomer subunit epsilon-1*, nothing found linked to plant adaptation to climate.*
- transcription factor PCL1, interact with SWI/SNF chromatin remodeling factors which interact with ABA signaling to establish plant stress memory (Bulgakov *et al.*, 2019).

- ALA-interacting subunit 3, *nothing found linked to plant adaptation to climate.*
- zinc finger CCHC domain-containing 8 isoform X2, *nothing found linked to plant adaptation to climate.*
- transcription termination factor chloroplastic-like, *nothing found linked to plant adaptation to climate.*
- pentatricopeptide repeat-containing At1g20230, *nothing found linked to plant adaptation to climate.*
- cyclin-A1-4 isoform X1, *nothing found linked to plant adaptation to climate.*
- DNA topoisomerase 2, *nothing found linked to plant adaptation to climate.*
- BP28CT domain-containing, *nothing found linked to plant adaptation to climate.*
- U3snoRNP10 domain-containing, *nothing found linked to plant adaptation to climate.*
- carboxyl-terminal-processing peptidase chloroplastic, *nothing found linked to plant adaptation to climate.*
- peptidyl-prolyl cis-trans isomerase Pin1, *nothing found linked to plant adaptation to climate.*
- hypothetical protein L484_013434, *nothing found linked to plant adaptation to climate.*
- E3 ubiquitin- ligase MARCH8-like isoform X1
- probable galacturonosyltransferase-like 7
- probable E3 ubiquitin- ligase ARI8
- katanin p80 WD40 repeat-containing subunit B1 homolog isoform X1
- pentatricopeptide repeat-containing At5g04780-like
- 2-3 ethylene-responsive transcription factor, stress-responsive factors in plants (Thirugnanasambantham *et al.*, 2015).
- probable tRNA N6-adenosine mitochondrial isoform X2, *nothing found linked to plant adaptation to climate.*
- lactosylceramide 4-alpha-galactosyltransferase-like, *nothing found linked to plant adaptation to climate.*
- NAD(P)-binding rossmann-fold, *nothing found linked to plant adaptation to climate.*
- methyltransferase 6, methylation can play a role in plant response to stress (Bartels *et al.*, 2018) and noticeably for conifers (Prunier *et al.*, 2016).
- signal recognition particle 19 kDa, *nothing found linked to plant adaptation to climate.*

- cytochrome b5 domain-containing RLF*, nothing found linked to plant adaptation to climate.*
- transmembrane 161B*, nothing found linked to plant adaptation to climate.*
- LAG1 longevity assurance homolog 3-like, potentially involved in pathogen resistance (Brandwagt *et al.*, 2000).
- tRNA wybutosine-synthesizing 2 3 4 isoform X1*, nothing found linked to plant adaptation to climate.*
- Suppressor of npr1 constitutive 1-like (SNC1) isoform X2, disease resistance (Mang *et al.*, 2012).
- pentatricopeptide repeat-containing mitochondrial, mediate gene expression (Manna 2015)
- U-box domain-containing 44-like, plays a role in senescence regulation and interact with ABA signaling (Raab *et al.*, 2009).
- disease resistance RPP13 4, involved in pathogen resistance in *Arabidopsis thalinana.*
- probable ubiquitin-conjugating enzyme E2 23*, nothing found linked to plant adaptation to climate.*
- ribosomal lysine N-methyltransferase 3-like isoform X1*, nothing found linked to plant adaptation to climate.*
- 5-formyltetrahydrofolate cycloligase*, nothing found linked to plant adaptation to climate.*
- probable phosphatase 2C 59 isoform X1*, nothing found linked to plant adaptation to climate.*
- pyruvate dehydrogenase (acetyl-transferring) mitochondrial*, nothing found linked to plant adaptation to climate.*
- H ACA ribonucleo complex subunit 2*, nothing found linked to plant adaptation to climate.*
- thioredoxin chloroplastic-like, plays a role in light signaling and photosynthesis (Nikkanen & Rintamäki, 2019).
- TITAN isoform X1*, nothing found linked to plant adaptation to climate.*
- GRIP*, nothing found linked to plant adaptation to climate.*
- pentatricopeptide repeat-containing At2g13600-like*, nothing found linked to plant adaptation to climate.*
- adenylylsulfatase HINT3 isoform X2*, nothing found linked to plant adaptation to climate.*
- Nucleolar pre-ribosomal-associated 1*, nothing found linked to plant adaptation to climate.*
- zinc finger 593*, nothing found linked to plant adaptation to climate.*

- translation elongation factor-1*, nothing found linked to plant adaptation to climate.*
- UDP glucose: glyco glucosyltransferase*, nothing found linked to plant adaptation to climate.*
- DNA topoisomerase 6 subunit B*, nothing found linked to plant adaptation to climate.*

CLUSTER 2

- rRNA methylase: methylation of ribosomal RNA is known to play a role in response to stress by enhancing or reducing translation of specific mRNAs (Liberman *et al.*, 2020)
- DNA damage-binding 1: involved in *Brassicaceae* adaptation to high altitude (Guo *et al.*, 2018).
- DHAR2 protein: involved in the production of ascorbate which is used by plant to survive under stress condition, for example high-light condition in *Arabidopsis thaliana* (Terai *et al.*, 2020).
- CHR4-like isoform, binds DNA through histones and regulates gene transcription
- ACD11 (accelerated cell death 11), overexpression of ACD11, triggered by an increase of ABA, improves salt and drought tolerance in *Arabidopsis thaliana* (Li, 2019).
- high mobility group B 15 (HMGB) isoform: high salinity, drought and cold tolerance in *Arabidopsis thaliana* (Kwak *et al.*, 2007).
- TPR2 (TOPLESS-RELATED PROTEIN) isoform, involved in biotic and abiotic stress response in *Arabidopsis thaliana* and potentially involved in flowering too (Causier *et al.*, 2012).
- Duplicated homeodomain-like superfamily isoform (transcription factor): involved in drought stress in poplars (Yoon *et al.*, 2014).
- apoptotic chromatin condensation inducer in the nucleus
- probable L-type lectin-domain containing receptor kinase
- pentatricopeptide repeat-containing chloroplastic-like (PPR): large family of proteins which have been found involved in the response of different biotic and abiotic stress response in poplar (Xing *et al.*, 2018).
- Mediator of ABA-Regulated Dormancy 1 (MARD1): ABA is a hormone involved in initiating drought stress response in trees (Hamanishi & Campbell, 2011).
- B-cell receptor-associated*, nothing found linked to plant adaptation to climate.*
- pectate lyase*, nothing found linked to plant adaptation to climate.*
- serine threonine- kinase pakA-like*, nothing found linked to plant adaptation to climate.*

- beta-galactosidase 8, associated with cold tolerance in citrus (Lang *et al.*, 2005).
- D-tagatose-1,6-bisphosphate aldolase subunit kbaZ*, nothing found linked to plant adaptation to climate.*
- probable LRR receptor-like serine threonine- kinase At1g56140*, nothing found linked to plant adaptation to climate.*
- anaphase-promoting complex subunit 2*, nothing found linked to plant adaptation to climate.*

REFERENCES

Bartels A, Han Q, Nair P, Stacey L, Gaynier H, Mosley M, Huang QQ, Pearson JK, Hsieh TF, An YQC, *et al.* 2018. Dynamic DNA methylation in plant growth and development. *International Journal of Molecular Sciences* **19**: 2144.

Behringer D, Zimmermann H, Ziegenhagen B, Liepelt S. **2015**. Differential gene expression reveals candidate genes for drought stress response in Abies alba (Pinaceae). *PLoS ONE* **10**: 1–18.

Bonardi V, Tang S, Stallmann A, Roberts M, Cherkis K, Dangl JL, Bonardi V, Tang S, Stallmann A, Roberts M, *et al.* 2017. Correction: Expanded functions for a family of plant intracellular immune receptors beyond specific recognition of pathogen effectors (Proceedings of the National Academy of Sciences of the United States of America (2011) 108 (16463-16468) DOI: 10.1073/p. *Proceedings of the National Academy of Sciences of the United States of America* **114**: E108.

Brandwagt BF, Mesbah LA, Takken FLW, Laurent PL, Kneppers TJA, Hille J, Nijkamp HJJ. **2000**. A longevity assurance gene homolog of tomato mediates resistance to Altemaria alternata f. sp. lycopersici toxins and fumonisin B1. *Proceedings of the National Academy of Sciences of the United States of America* **97**: 4961–4966.

Bulgakov VP, Wu HC, Jinn TL. **2019**. Coordination of ABA and Chaperone Signaling in Plant Stress Responses. *Trends in Plant Science* **24**: 636–651.

Causier B, Ashworth M, Guo W, Davies B. **2012**. The TOPLESS interactome: A framework for gene repression in Arabidopsis. *Plant Physiology* **158**: 423–438.

Guo X, Hu Q, Hao G, Wang X, Zhang D, Ma T, Liu J. **2018**. The genomes of two Eutrema species provide insight into plant adaptation to high altitudes. *DNA Research* **25**: 307–315.

Guo M, Liu JH, Ma X, Luo DX, Gong ZH, Lu MH. **2016**. The plant heat stress transcription factors (HSFS): Structure, regulation, and function in response to abiotic stresses. *Frontiers in Plant Science* **7**: 114.

Hamanishi ET, Campbell MM. **2011**. Genome-wide responses to drought in forest trees. *Forestry* **84**: 273–283.

Kim HS, Park W, Lee HS, Shin JH, Ahn SJ. **2021**. Subcellular Journey of Rare Cold Inducible 2 Protein in Plant Under Stressful Condition. *Frontiers in Plant Science* **11**: 1–11.

Kosmacz M, Luzarowski M, Kerber O, Leniak E, Gutiérrez-Beltrán E, Moreno JC, Gorka M, Szlachetko J, Veyel D, Graf A, *et al.* **2018**. Interaction of 2',3'-cAMP with Rbp47b Plays a Role in Stress Granule Formation. *Plant physiology* **177**: 411–421.

Kwak KJ, Kim JY, Kim YO, Kang H. **2007**. Characterization of transgenic arabidopsis plants overexpressing high mobility group B proteins under high salinity, drought or cold stress. *Plant and Cell Physiology* **48**: 221–231.

Lang P, Zhang CK, Ebel RC, Dane F, Dozier WA. **2005**. Identification of cold acclimated genes in leaves of Citrus unshiu by mRNA differential display. *Gene* **359**: 111–118.

Li Q. **2019**. Identification of Roles for Arabidopsis thaliana RING-Type Ubiquitin Ligase XBAT35.2 and its Substrate Accelerated Cell Death11 (ACD11) in Abiotic Stress Tolerance. *Doctoral dissertation*. http://hdl.handle.net/10222/80180.

Liberman N, O'Brown ZK, Earl AS, Boulias K, Gerashchenko MV, Wang SY, Fritsche C, Fady PE, Dong A, Gladyshev VN, *et al.* **2020**. N6-adenosine methylation of ribosomal RNA affects lipid oxidation and stress resistance. *Science Advances* **6**: eaaz4370.

Luo, Y., Wang, Z., Ji, H., Fang, H., Wang, S., Tian, L., & Li, X. 2013. An A rabidopsis homolog of importin β1 is required for ABA response and drought tolerance. *The Plant Journal* **75**: 377-389.

Manna, S. 2015. An overview of pentatricopeptide repeat proteins and their applications. *Biochimie* **113**: 93-99.

Mang HG, Qian W, Zhu Y, Qian J, Kang HG, Klessig DF, Hua J. **2012**. Abscisic acid deficiency antagonizes high-temperature inhibition of disease resistance through enhancing nuclear accumulation of resistance proteins SNC1 and RPS4 in Arabidopsis. *Plant Cell* **24**: 1271–1284.

Mazzucotelli E, Belloni S, Marone D, De Leonardis A, Guerra D, Di Fonzo N, Cattivelli L, Mastrangelo A. **2006**. The E3 Ubiquitin Ligase Gene Family in Plants: Regulation by Degradation. *Current Genomics* **7**: 509–522.

Nikkanen L, Rintamäki E. **2019**. Chloroplast thioredoxin systems dynamically regulate photosynthesis in plants. *Biochemical Journal* **476**: 1159–1172.

Prunier J, Verta JP, Mackay JJ. **2016**. Conifer genomics and adaptation: At the crossroads of genetic diversity and genome function. *New Phytologist* **209**: 44–62.

Raab S, Drechsel G, Zarepour M, Hartung W, Koshiba T, Bittner F, Hoth S. **2009**. Identification of a novel E3 ubiquitin ligase that is required for suppression of premature senescence in Arabidopsis. *Plant Journal* **59**: 39–51.

Schomburg FM, Patton DA, Meinke DW, Amasino RM. **2001**. FPA, a gene involved in floral induction in Arabidopsis, encodes a protein containing RNA-recognition motifs. *Plant Cell* **13**: 1427–1436.

Shi J, Du X. **2020**. Identification, characterization and expression analysis of calmodulin and calmodulin-like proteins in Solanum pennellii. *Scientific Reports* **10**: 1–17.

Sung S, Schmitz RJ, Amasino RM. **2006**. A PHD finger protein involved in both the vernalization and photoperiod pathways in Arabidopsis. *Genes and Development* **20**: 3244–3248.

Terai Y, Ueno H, Ogawa T, Sawa Y, Miyagi A, Kawai-Yamada M, Ishikawa T, Maruta T. **2020**. Dehydroascorbate reductases and glutathione set a threshold for high-light–induced ascorbate accumulation. *Plant Physiology* **183**: 112–122.

Thirugnanasambantham K, Durairaj S, Saravanan S, Karikalan K, Muralidaran S, Islam VIH. **2015**. Role of Ethylene Response Transcription Factor (ERF) and Its Regulation in Response to Stress Encountered by Plants. *Plant Molecular Biology Reporter* **33**: 347–357.

Tornero P, Merritt P, Sadanandom A, Shirasu K, Innes RW, Dangl JL. **2002**. RAR1 and NDR1 contribute quantitatively to disease resistance in Arabidopsis, and their relative contributions are dependent on the R gene assayed. *Plant Cell* **14**: 1005–1015.

Xing H, Fu X, Yang C, Tang X, Guo L, Li C, Xu C, Luo K. **2018**. Genome-wide investigation of pentatricopeptide repeat gene family in poplar and their expression analysis in response to biotic and abiotic stresses. *Scientific Reports* **8**: 1–9.

Yoon SK, Park EJ, Choi YI, Bae EK, Kim JH, Park SY, Kang KS, Lee H. **2014**. Response to drought and salt stress in leaves of poplar (Populus alba×Populus glandulosa): Expression profiling by oligonucleotide microarray analysis. *Plant Physiology and Biochemistry* **84**: 158–168.

Zhao Y, Li X, Wang F, Zhao X, Gao Y, Zhao C, He L, Li Z, Xu J. **2018**. Glycerol-3-phosphate dehydrogenase (GPDH) gene family in Zea mays L.: Identification, subcellular localization, and transcriptional responses to abiotic stresses. *PLoS ONE* **13**: 1–20.

Zhou J, Wang J, Cheng Y, Chi YJ, Fan B, Yu JQ, Chen Z. **2013**. NBR1-Mediated Selective Autophagy Targets Insoluble Ubiquitinated Protein Aggregates in Plant Stress Responses. *PLoS Genetics* **9**: e1003196.
